# Description of *Triatoma mopan* sp. n. from a cave in Belize (Hemiptera, Reduviidae, Triatominae)

**DOI:** 10.3897/zookeys.775.22553

**Published:** 2018-07-17

**Authors:** Patricia L. Dorn, Silvia A. Justi, Carolina Dale, Lori Stevens, Cleber Galvão, Raquel Lima-Cordón, Carlota Monroy

**Affiliations:** 1 Department of Biological Sciences, Loyola University New Orleans, New Orleans, LA, USA; 2 Department of Biology, University of Vermont, Burlington, VT, USA; 3 Laboratório de Biodiversidade Entomológica, Instituto Oswaldo Cruz, FIOCRUZ, Rio de Janeiro, Brazil; 4 Laboratório Nacional e Internacional de Referência em Taxonomia de Triatomíneos, Instituto Oswaldo Cruz, FIOCRUZ, Rio de Janeiro, Brazil; 5 LENAP, University of San Carlos, Guatemala City, Guatemala; 6 The Walter Reed Biosystematics Unit, Smithsonian Institution Museum Support Center, 4210 Silver Hill Rd, Suitland, MD 20746-2863, USA

**Keywords:** Belize, Chagas disease, new species, *Triatoma dimidiata*, *Triatoma mopan*

## Abstract

In this paper, *Triatoma mopan* **sp. n.** is described based on five males and six females collected in the Rio Frio cave, Cayo District, Belize. This species is similar to *Triatoma dimidiata* (Latreille), but can be distinguished by characters found on the pronotum, legs, and abdomen. Geometric morphometry and phylogenetic comparisons are also provided. Presently, the species is known only from the type locality and is a potential Chagas vector.

## Introduction

Species belonging to Triatominae Jeannel, 1919 (Insecta, Hemiptera, Reduviidae) are important as vectors of Chagas disease. Presently, more than 150 species within 15 genera (Justi and Galvão 2017) are recognized as valid in this subfamily. The most speciose genus is *Triatoma* Laporte, 1832, which includes *Triatoma dimidiata* (Latreille, 1811) and *Triatoma infestans* (Klug, 1834), historically, two of the most relevant Chagas disease vectors in Central and South America, respectively.

Because of the substantial morphological variation of *T. dimidiata* across its large geographic distribution (southern Mexico to Peru), this species has been split and synonymized many times since its original description (reviewed in Dorn et al. 2007). This includes morphotypes (previously considered species or subspecies) at extreme ends of the geographic distribution. In the north (Mexico, described as *T. maculipennis* Stål, 1859), specimens of *T. dimidiata* have smaller bodies, shorter heads, and larger eyes compared with specimens from the south (Colombia, once called *T. capitata* Usinger 1941).

Lent and Wygodzinsky (1979) examined 160 *T. dimidiata* specimens, including the entire distribution, and concluded that, in general, there is a clinal variation with size increasing southwards, and synonymized *T. maculipennis* and *T. capitata* with *T. dimidiata*; these authors also state that there are many exceptions to that rule, and commented specifically on cave specimens. Upon comparison of one “*T. dimidiata*” specimen from the Rio Frio cave, in Belize, with five specimens from the Lanquin cave in Guatemala, the authors conclude that they appear identical. The morphological distinctions between the cave specimens and the other specimens of *T. dimidiata* were regarded simply as cave adaptations.

Following the synonymizing of the species (Lent and Wygodzinsky 1979), many qualitative and quantitative phenotypic, biochemical, and molecular studies have sought to clarify the systematics of *T. dimidiata*. Phylogenetic studies of *T. dimidiata* based on DNA sequence analyses of nuclear and mitochondrial genes (Bargues et al. 2008, Monteiro et al 2003, Dorn et al. 2016) and genome-wide data (Justi et al. 2018) have shown that *T. dimidiata* is composed of at least three phylogenetic species (Mishler and Brandon 1987), referred to as : *T. dimidiata* sensu strictu (or groups 1 and 2), *T.* sp. *affinnis dimidiata* (group 3) and T. sp. aff. dimidiata – cave (group 4); the latter comprises only specimens from the Rio Frio cave, Cayo District, Belize. Interestingly, Dorn et al. (2016) included in their study specimens from both caves mentioned in Lent and Wygodzinsky (1979) and found that specimens from Lanquin are recovered within *T. dimidiata* s.s., whereas the ones from Rio Frio compose a distinct clade.

In this manuscript, we describe T. sp. aff. dimidiata – cave, the lineage from the Rio Frio cave, as *Triatoma mopan* sp. n. (Hemiptera, Reduviidae, Triatominae), a new species of the genus *Triatoma*.

## Materials and methods

### Specimen collection

We conducted field work on June 15, 2016 in the Rio Frio cave, Cayo District, Belize [(coordinates: 16.956939/-88.979675) under permits covering the research (#IA/H/1/16 (03), Institute of Archaeology), collecting (#WL/1/1/16 (33), Forest Department) and export (#WL/1/7/16 (29), Forest Department) of specimens from Belize. The sole purpose of this field work was to collect enough specimens from the Rio Frio cave *Triatoma* population to reliably compare this population with *T. dimidiata* from other localities. We collected specimens from the Rio Frio cave, Cayo, Belize because of previous results of phylogenetic studies that showed this population to be an independent lineage distinct from all other populations included under the umbrella of *T. dimidiata*, and to be the only phylogenetic species found in this particular cave. We collected 15 adult males and 13 adult females and more than 70 nymphs of various lifecycle stages. For this study, we focus on the adult morphology.

### Morphological identification and description

Adults collected in the Rio Frio cave could not be taxonomically identified using the key for *Triatoma* species (Lent and Wygodzinsky 1979). Because of the morphological similarities mentioned by Lent and Wygodzinsky (1979) between *T. dimidiata* and the single specimen they examined from the Rio Frio cave, six *T. mopan* males and seven *T. mopan* females were compared with specimens from the Lanquin cave, Guatemala, as well as representatives of the entire distribution of *T. dimidiata*, including photographs of the type specimens from the extreme ends of the range, *T. dimidiata capitata* (from Colombia), and *T. dimidiata maculipennis* (from Mexico). It was not possible to directly compare with the *T. dimidiata* holotype, since this specimen has been lost; thus, *T. dimidiata* specimens used in this study were identified following the key and description in Lent and Wygodzinsky (1979). The specimens examined here were from the Triatominae collection of the Oswaldo Cruz Institute (CTIOC) in Rio de Janeiro, Brazil (Table 1) and the type specimens were from Zoologisches Museum, Berlin (*T. dimidiata maculipennis*) and the California Academy of Sciences, USA (*T. dimidiata capitata*).

**Table 1.** Specimens used for morphological and morphometric comparison. CTIOC: Triatominae Collection of the Oswaldo Cruz Institute.

Species	Specimen origin	ID/voucher number	Geographic Origin	Sex	Notes
T. sp. aff. dimidiata	field	A10800	Huehuetenango, Guatemala	F	
	field	A10727	Huehuetenango, Guatemala	M	
*Triatoma dimidiata* *s.l.*	CTIOC	2838	Colombia	M	capitata morphotype
	Colony LNIRTT*	N/A	Colombia	F	capitata morphotype
	CTIOC	N/A	Santa Boyaca, Colombia	F	capitata morphotype
	CTIOC	2463	Costa Rica	F	
	CTIOC	2592	Costa Rica	F	
	CTIOC	2587	San Jose, Costa Rica	F	
	Colony LNIRTT*	N/A	Equador	F	
	Colony LNIRTT*	N/A	Equador – genitalia	M	
	CTIOC	3385	Candelaria Caves, Alta Verapaz, Guatemala	M	
	CTIOC	A6160	Lanquin Caves, Guatemala	M	
	CTIOC	3388	Lanquin Caves, Guatemala	M	
	CTIOC	3377	Peten	M	
	CTIOC	3379	Peten	F	
	CTIOC	A9703	Peten	F	
	CTIOC	8937	Mexico	M	maculipennis morphotype
	CTIOC	N/A	Peru	M	
	CTIOC	2769	N/A	M	
	California Academy of Sciences	N/A	Boyacá, Colombia	M	capitata holotype
*Triatoma gerstaeckeri*	CTIOC	6242	Mexico	F	
	CTIOC	N/A	San Marcos, Texas, US	M	
	CTIOC	N/A	Texas	M	
	CTIOC	6239	N/A	F	
	CTIOC	6241	N/A	F	
*Triatoma mopan*	Colony 16 LNIRTT	N/A	Belize	F	colony started in 12/05/2006
	Colony 147 LNIRTT	N/A	Belize	F	colony started in 12/05/2006
	Field	2016BZ001	Cayo District, Rio Frio Cave,	F	
	Field	2016BZ002	Cayo District, Rio Frio Cave, Belize	F	
	Field	2016BZ003	Cayo District, Rio Frio Cave, Belize	F	
	Field	2016BZ004	Cayo District, Rio Frio Cave, Belize	F	
	Field	2016BZ005	Cayo District, Rio Frio Cave, Belize	F	
	Field	2016BZ006	Cayo District, Rio Frio Cave, Belize	F	
	Field	2016BZ007	Cayo District, Rio Frio Cave, Belize	M	
	Field	2016BZ008	Cayo District, Rio Frio Cave, Belize	M	
	Field	2016BZ009	Cayo District, Rio Frio Cave, Belize	M	
	Field	2016BZ011	Cayo District, Rio Frio Cave, Belize	M	
	Field	2016BZ013	Cayo District, Rio Frio Cave, Belize	M	

### Morphological study

Character observation and measurements were made with a stereoscopic Zeiss Stemi SV11 microscope, using a graduated eyepiece micrometer, and photos were taken using a Nikon Coolpix 990 digital camera. The following characters were measured:

**TL** total length of the body

**LOP** and **WOP** length and width of pronotum

**AOR** and **POR** length of the ante- and post-ocular region

**SYN** length of the inter-ocular region or synthlipsis

**HL** and **WOH** length and width of the head

**WE** width of the eye

**TS** total length of scutellum

**POS** length of process of scutellum

**A1**-**A4** length of antennal segments

**Figure 1.**
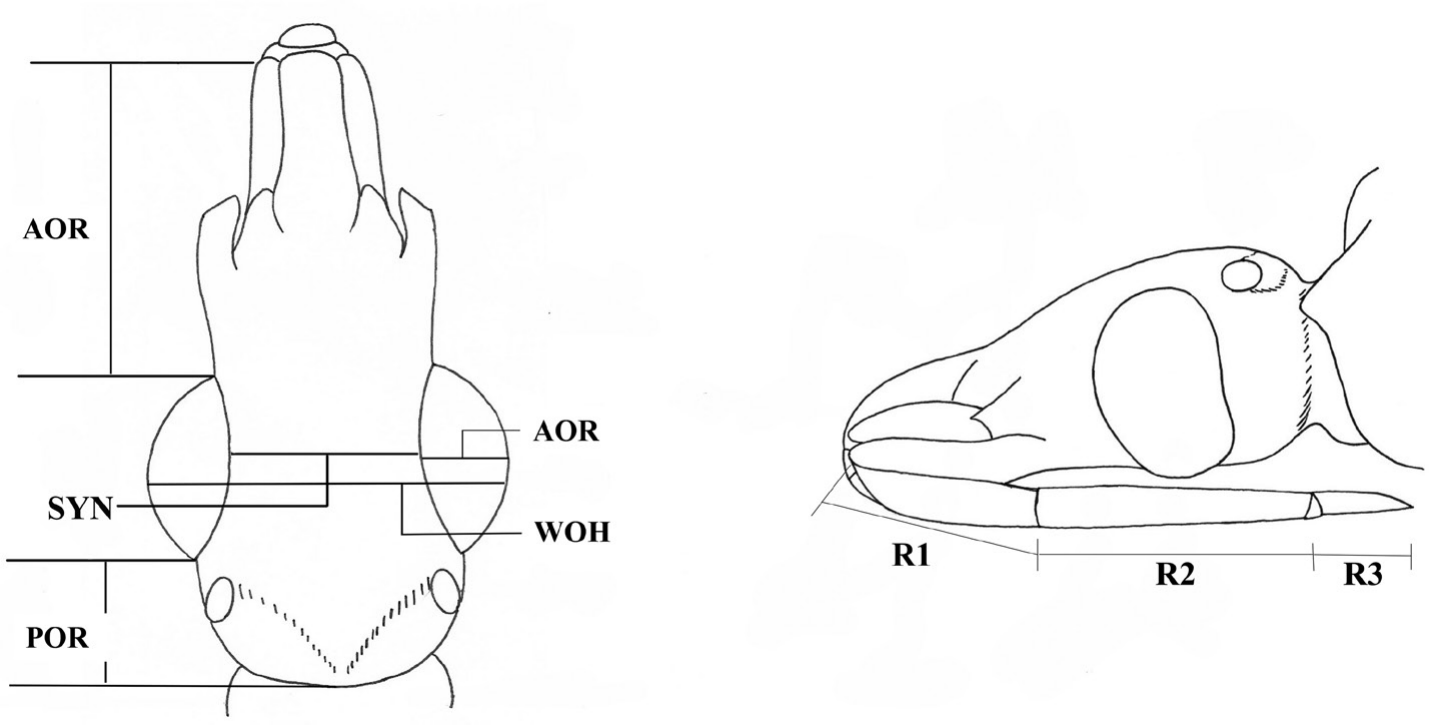
Scheme of the characters measured on the head.

**R1-R3** labial segments (Figure 1, Table 2).

The terminology and measurements used for the description were based on Lent and Wygodzinsky (1979). For comparison, these characters were also measured for specimens of *T. dimidiata*. For each sex separately, the two species were compared for each of the characters with a t-test using JMP® ver 13 (SAS Institute, Inc., Cary NC, USA). Significance was adjusted for multiple comparisons with the method of Benjamini and Hochberg (1995). Boxplots comparing the characters measured were also created using R (The R Development Core Team 2008) and the code and plots are provided as Suppl. material 1.

**Table 2.** Sequence information and specimens used for the phylogenetic reconstruction and calculation of genetic distances.

Taxon	Sequence ID	Locality	ITS-2	Cyt b
*T. dimidiata*	1	Sta. Theresa, Toledo	DQ871354	FJ197155
	10	Caserío la Bendición, Monte Largo, Santa Ana, El Salvador	AM286693	JN585881
	11	Caserío la Bendición, Monte Largo, Santa Ana, El Salvador	AM286693	JN585881
	12	Caserío la Bendición, Monte Largo, Santa Ana, El Salvador	AM286693	JN585881
	13	Caserío la Bendición, Monte Largo, Santa Ana, El Salvador	AM286695	JN585881
	14	Caserío la Bendición, Monte Largo, Santa Ana, El Salvador	KT874438	JN585881
	15	Sto. Tomás, Sto. Domingo, Heredia, Costa Rica	AM286693	JN585893
	16	Sto. Tomás, Sto. Domingo, Heredia, Costa Rica	AM286693	JN585894
	17	Sto. Tomás, Sto. Domingo, Heredia, Costa Rica	AM286693	JN585894
	18	Angeles, San Rafael, Heredia, Costa Rica	KF192843	JN585894
	19	Sto. Tomás, Sto. Domingo, Heredia, Costa Rica	KT874433	JN585895
	2	Mérida, Yucatán, Mexico	FJ197146	FJ197157
	20	Colombia	AM286703	KT998309
	21	Colombia	AM286703	KT998309
	22	Colombia	AM286704	KT998309
	23	Colombia	KF192845	KT998310
	24	Lanquin, Alta Verapaz, Guatemala	AM286702	KT998313
	25	Lanquin, Alta Verapaz, Guatemala	AM286702	KT998314
	26	El Lodo Negro, San Marcos Sierra, Intibuca, Honduras	AM286694	KT998315
	27	El Masical, San Antonio, Copán, Honduras	AM286694	KT998316
	28	El Masical, San Antonio, Copán, Honduras	AM286695	KT998316
	29	Caserío la Bendición, Monte Largo, Santa Ana, El Salvador	AM286693	KT998317
	3	Lanquin, Alta Verapaz, Guatemala	AM286694	JN585861
	30	Caserío la Bendición, Monte Largo, Santa Ana, El Salvador	AM286696	KT998318
*T. dimidiata*	31	El Lodo Negro, San Marcos Sierra, Intibuca, Honduras	AM286695	KT998319
	32	El Masical, San Antonio, Copán, Honduras	AM286694	KT998320
	33	El Lodo Negro, San Marcos Sierra, Intibuca, Honduras	AM286693	KT998321
	34	El Lodo Negro, San Marcos Sierra, Intibuca, Honduras	KT874435	KT998321
	35	El Lodo Negro, San Marcos Sierra, Intibuca, Honduras	KT874437	KT998321
	36	El Masical, San Antonio, Copán, Honduras	AM286693	KT998322
	37	El Masical, San Antonio, Copán, Honduras	KT874436	KT998322
	38	El Lodo Negro, San Marcos Sierra, Intibuca, Honduras	AM286693	KT998325
	39	El Lodo Negro, San Marcos Sierra, Intibuca, Honduras	AM286694	KT998325
	4	Lanquin, Alta Verapaz, Guatemala	AM286702	JN585861
	40	El Lodo Negro, San Marcos Sierra, Intibuca, Honduras	AM286695	KT998325
	41	El Masical, San Antonio, Copán, Honduras	KT874434	KT998325
	42	Caserío la Bendición, Monte Largo, Santa Ana, El Salvador	AM286693	KT998327
	43	Angeles, San Rafael, Heredia, Costa Rica	AM286693	KT998328
	44	Sto. Tomás, Sto. Domingo, Heredia, Costa Rica	KT874432	KT998330
	45	Angeles, San Rafael, Heredia, Costa Rica	KF192844	KT998331
	46	San Pedro Columbia, Toledo district, Belize	FJ197153	FJ197154
	5	Caserío la Bendición, Monte Largo, Santa Ana, El Salvador	AM286693	JN585881
	6	Caserío la Bendición, Monte Largo, Santa Ana, El Salvador	AM286693	JN585881
	7	Caserío la Bendición, Monte Largo, Santa Ana, El Salvador	AM286693	JN585881
	8	Caserío la Bendición, Monte Largo, Santa Ana, El Salvador	AM286693	JN585881
	9	Caserío la Bendición, Monte Largo, Santa Ana, El Salvador	AM286693	JN585881
T. sp. aff dimidiata	1	Calla Creek, Cayo District, Belize	FJ197152	FJ197156
	2	Mérida, Yucatán, Mexico	FJ197150	FJ197158
	3	Mérida, Yucatán, Mexico	FJ197147	FJ197159
	4	Teya, Yucatán, Mexico	KT874439	KT998296
	5	Huehuetenango, Guatemala		
	5	Huehuetenango, Guatemala		
*T. mopan*	1	Río Frio Cave, Cayo District, Belize	KF192846	JN585883
	2	Río Frio Cave, Cayo District, Belize	KF192847	JN585884
	Col16			
*T. infestans*			AJ576054	JN006799
*T. gerstaeckeri*		Chihuahua	JQ282707	JQ282723
*T. brailovskyi*		Jalisco	JQ282704	JQ282720
*T. hegneri*			AM286727	JN585830

### Morphometric study

**Figure 2.**
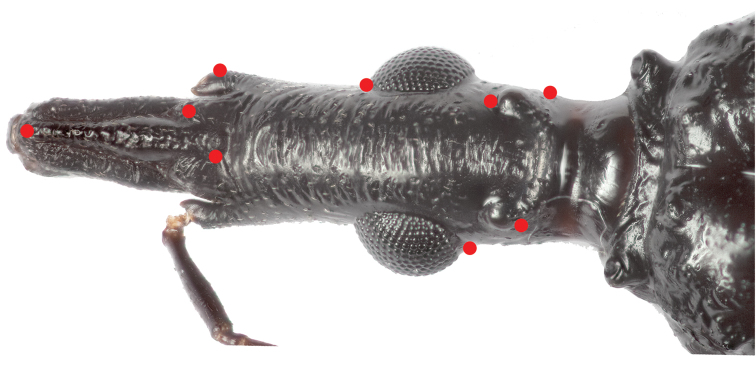
Landmarks selected for the morphometric comparison. Photograph Raquel Lima.

Morphometric comparison of the dorsal portion of the heads was made based on eight landmarks (Figure 2). Thirteen *T. mopan* specimens (eleven from the field and two from a Laboratório Nacional e Internacional de Referência em Taxonomia de Triatomíneos – LNIRTT colony, previously regarded as *T. dimidiata* but found to be *T. mopan* based on DNA sequence (T. sp. aff. dimidiata – cave; Justi et al. 2018) were compared with all known morphotypes of *T. dimidiata* s.l. (*T. dimidiata maculipennis*: one specimen from Mexico; *T. dimidiata capitata*: four specimens including the holotype; *Triatoma dimidiata* s.l.: one each from Mexico and Peru, three from Costa Rica, two from Ecuador, six from Guatemala, including two from the Lanquin cave; T. sp. aff. dimidiata: two from Guatemala); and *T. gerstaeckeri*: five from the southern United States (Table 1). *Triatoma gerstaeckeri* was included because this species was recovered as a sister taxon to *T. mopan* (then identified as *T. dimidiata*) in a recent phylogenetic study (Justi et al. 2016). Size variation observed in colony specimens (Jaramillo et al. 2002) did not interfere with the analyses since for geometric morphometric analysis size information is removed. Additionally, colony specimens were not used for character (morphology) measurements.

In order to compare the head shape variation between *T. mopan* and closely related taxa, we used eight landmarks (clear, visible, homologous intersections between structures; Jaramillo 2011), taken for each specimen using StereoMorph (Olsen and Westneat 2015). *Triatoma dimidiata* specimens were grouped according to morphotype, and all comparisons were done amongst groups. Generalized Procrustes analysis was performed so shapes were directly comparable, without the effect of size and this was followed by Principal Component Analysis (PCA) and Canonical Variate Analysis (CVA). ANOVA statistics were used to compare variance between the group means for Centroid size (ANOVA) and shape (Procrustes ANOVA), with the null hypothesis that the means are not different among groups. All analyses were performed using the package MorphoJ (Klingenberg lab 2014).

### Phylogeny and genetic distance

Portions of the nuclear Internal transcribed spacer 2 (ITS-2) and mitochondrial cytochrome b (cytb) gene sequences from all nominal species belonging to the *dimidiata* species subcomplex and *species affinis* (*T. dimidiata*, T. sp. aff. dimidiata, *T. gerstaeckeri*, *T. brailovskyi*, *T. hegneri*, and *T. mopan*; Table 2; (Justi and Galvão 2017) were used for phylogenetic reconstruction and comparison of genetic distances, with *T. infestans* as outgroup. *Triatoma mopan* specimens used for this purpose were the same as described from Rio Frio cave, Cayo, Belize in Monteiro et al. (2013), Dorn et al. (2016), Justi et al. (2018), including two specimens from the LNIRTT colony. PCR and sequencing of these genes was performed as previously described (Dorn et al. 2016).

Sequences were aligned using MAFFT version 7 (Katoh and Standley 2013) for ITS-2, with the algorithm Q-INS-I and ClustalW (Larkin et al. 2007) implemented on MEGA v. 6 (Tamura et al. 2013) for cytb. JModeltest (Darriba et al. 2015) was used to assess the best fit model of evolution under AIC criterion. Maximum likelihood phylogenies were reconstructed independently for each marker using PhyML v.3.1 (Guindon and Gascuel 2003) with 100 bootstrap replicates. The best-fit model for ITS-2 was HKY+G and for cytb, TPM2uf+G.

In order to evaluate previously reported genetic distances (Monteiro et al. 2003, Monteiro et al. 2004) and assess comparable intra- and interspecific distances with previously reported data (K2-p cytb distances < 2% for interspecific comparisons), we used the package ape (Paradis et al. 2004), in R (The R Development Core Team 2008); the code is provided as Supp. material 1 (S2).

## Taxonomy

### Family Reduviidae Latreille, 1807

#### Subfamily Triatominae Jeannel, 1919

##### Genus *Triatoma* Laporte, 1832

###### Triatoma mopan

Taxon classificationAnimaliaHemipteraReduviidae

Dorn, Justi & Dale, 2018 sp. n.

http://zoobank.org/94C6EBF5-D78A-4294-97EC-AAD58AAE2769

Figure 6

####### Material.

**Holotype** Male. BELIZE: Cayo: Rio Frio Cave, coordinates: 16.956939/-88.979675, 15 June 2016, Dorn, Justi, Stevens, Monroy, CTIOC, FIOCRUZ. **Paratypes.** Five males and six females, Cayo: Rio Frio Cave, coordinates: 16.956939/-88.979675, 15 June 2016, Dorn, Justi, Stevens, Monroy (CTIOC; FIOCRUZ [four males and five females]; National Museum of Natural History, Smithsonian Institution [one male and one female])

####### Diagnosis.

**Figure 3.**
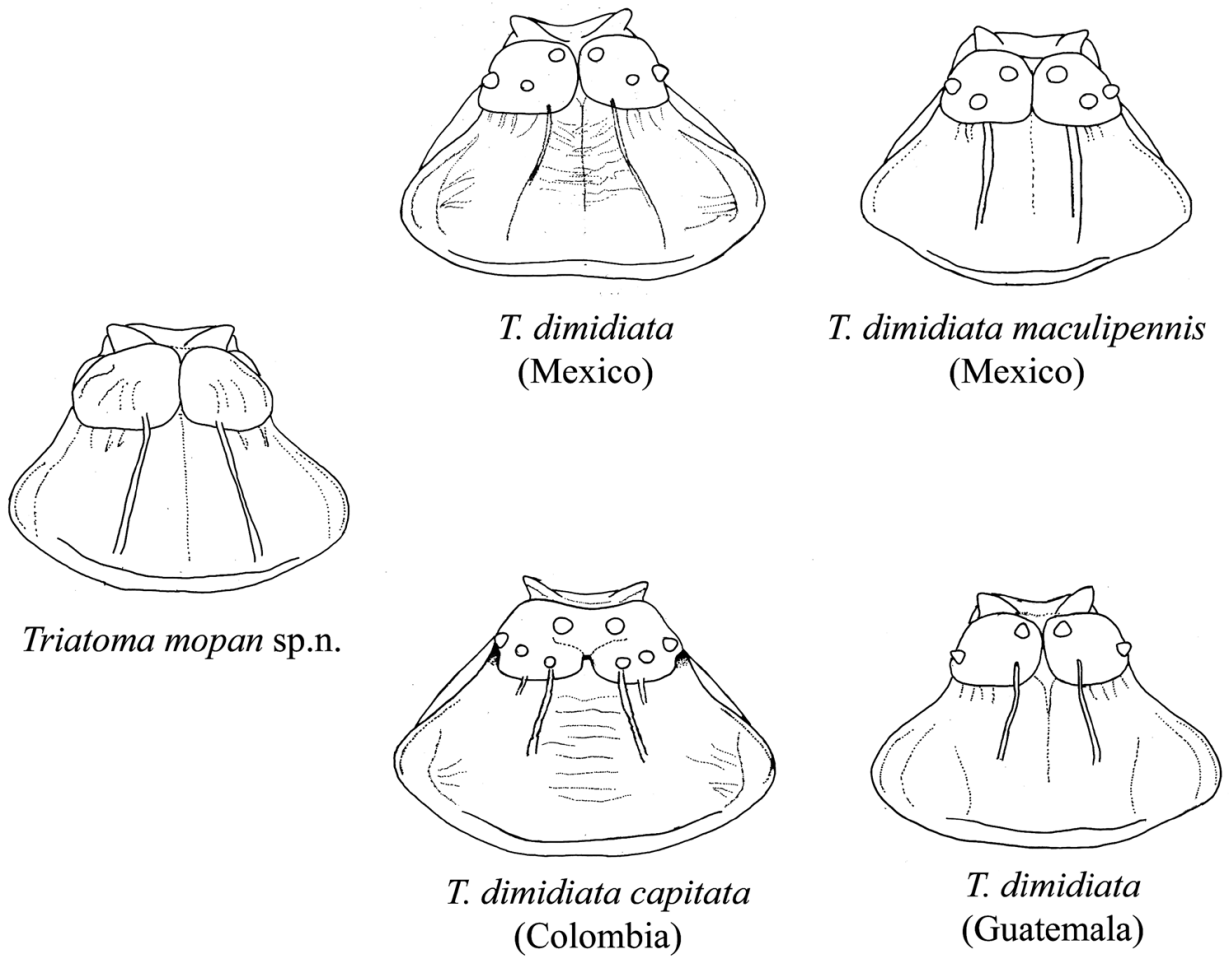
Drawings of the pronotum of *T. mopan* and distinct *T. dimidiata* morphotypes. Note that for *T. dimidiata* the anterior lobe shows discal and lateral tubercles, that are absent in *T. mopan*, which also presents a shorter and rounder anterolateral angle. Drawings by Carolina Dale.

**Figure 4.**
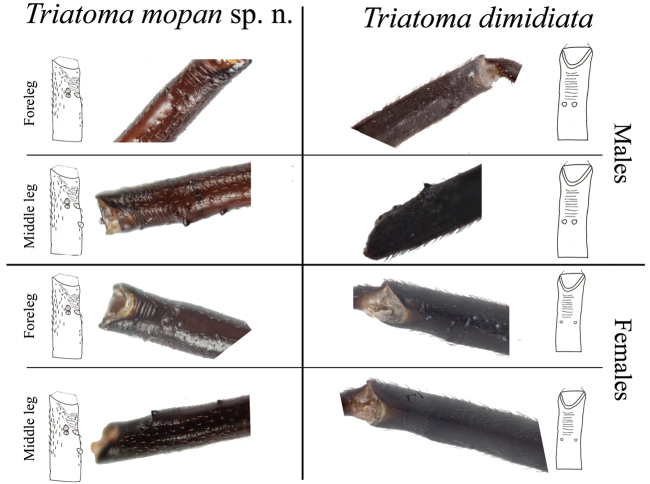
Comparison between the fore and mid femora of *T. mopan* and *T. dimidiata* from Jutiapa, Guatemala. Drawings: Carolina Dale. Photographs Silvia Justi.

**Figure 5.**
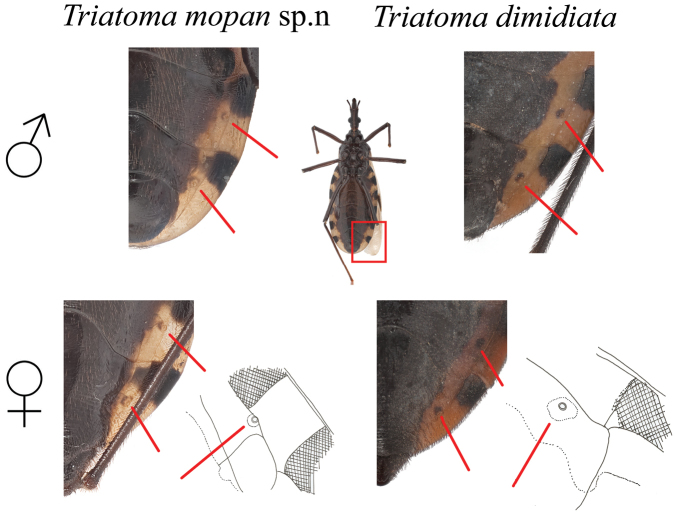
Comparison between the position and color of the abdominal spiracles from *T. mopan* and *T. dimidiata* from Jutiapa, Guatemala. Drawings by Carolina Dale. Photographs Silvia Justi and Raquel Lima.

*Triatoma mopan* has an overall vestiture similar to *T. dimidiata*, generally with a more slender body. It is believed to have been wrongly identified as *T. dimidiata* by Lent and Wygodzinsky (1979). Upon close examination, *T. mopan* can be easily distinguished from *T. dimidiata* specimens by the consistently pale-yellow hemelytra, and uniformly dark brown to black head and pronotum with scarce pale setae, absent in *T. dimidiata*. *Triatoma mopan* has the pronotum with a straight latitudinal depression dividing it in half and the anterior lobe of the pronotum rugose without any distinguishable tubercles (all the examined morphotypes of *T. dimidiata* present such tubercles) (Figure 3). In addition, the fore-femora with 1+1 apical, small denticles, 2 +1 subapical denticles in both males and females; and mid-femora with 1+1 apical, small denticles, 2 +2 asymmetrical subapical larger denticles on males and 2 +2 larger, asymmetrical subapical denticles on females. In contrast, *T. dimidiata* presents 1+1 small subapical denticles on both fore- and mid-femora in both sexes (Figure 4). Abdominal spiracles are close or adjacent to the connexival suture and surrounded by spot slightly darker then the tegument in *T. mopan*, but never adjacent and always surrounded by a well-defined dark spot in *T. dimidiata* (Figure 5).

####### Description.

**Figure 6.**
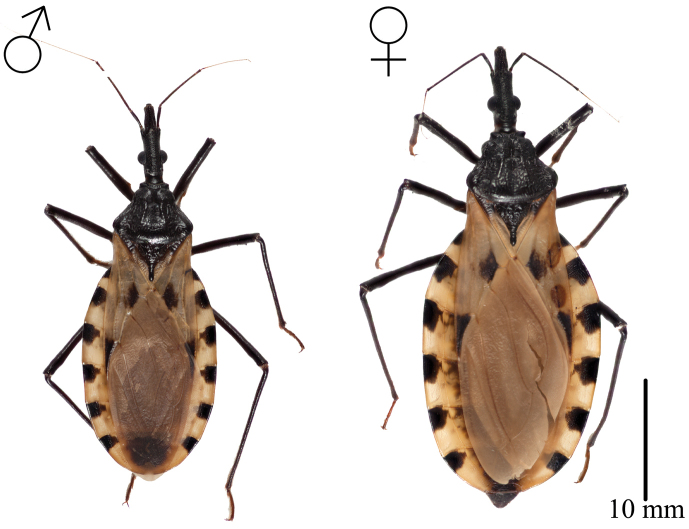
Overall vestiture of *T. mopan* male and female. Scalebar 10 mm. Photographs Raquel Lima.

Length of male 26.6–30.1 mm., of female 32.1–38 mm.; width of pronotum of male 6.2–6.3 mm., of female 6–7.4 mm (Table 4). Overall color dark brown or black, with connexivum and corium pale yellow. Pilosity pale yellow, short and scarce (Figure 6).

**Figure 7.**
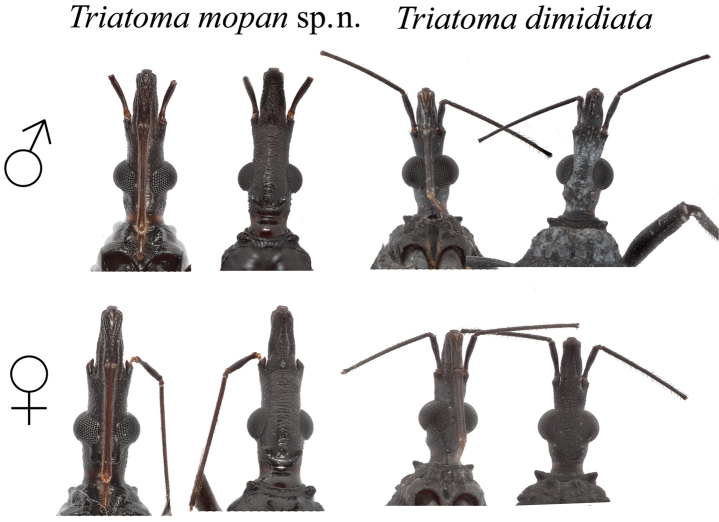
Comparison between the dorsal and ventral portions of the heads from *T. mopan* and *T. dimidiata* from Jutiapa, Guatemala males and females. Photographs: Silvia Justi and Raquel Lima.

*Head* distinctively rugose dorsally (Figure 7), averaging twice as long as width across the eyes (1:0.39–0.46) and slightly longer than the pronotum (1:1.09–1.23). Short, scarce, pale yellow pilosity present in both dorsal and ventral portions (Figure 7). Anteocular region from 2.8 to 3.2 times as long as post ocular (1:0.31–0.35), post ocular region with sides almost straight, subparallel and not rounded. Eyes in lateral view surpassing the level of the ventral surface of the head. Ratio of the width of eye to synthlipsis: 1:1.81–2.46. Ocelli very small, with diameter averaging about half the distance from their anterior border to the posterior margin of the eye. Antenniferous tubercles subcylindrical, situated slightly posterior of middle of anteocular region of head; first antennal segment attaining to the level of or surpassing the apex of clypeus; second segment with many strong setae. Antennal segments presenting a darker to lighter coloration from the first to the fourth segment, going from dark brown (first segment), reddish brown (second segment) to pale yellow (third and fourth segments). Ratio of antennal segments: 1:2.71–3.4:2.5–2.6:1.15. Labium slender; first visible segment extending almost to the base of the antenniferous tubercle in males and to the level of apex of antenniferous tubercle in females; second visible segment extending to the anterior portion of the thorax, almost reaching the stridulatory sulcus in males, and attaining level of posterior border of head on females; third visible segment attaining almost to the posterior portion of the stridulatory sulcus in males, and to the anterior half of the stridulatory sulcus on females (Figure 7). Ratio of visible labium segments: 1:1.79–2.15:0.44–0.69. Color dark brown (first visible segment) to light reddish brown (third visible segment). Setae pale yellow, with length and density increasing from the first visible through the third visible segment, being the longest and denser on the third segment (Figure 7).

**Figure 8.**
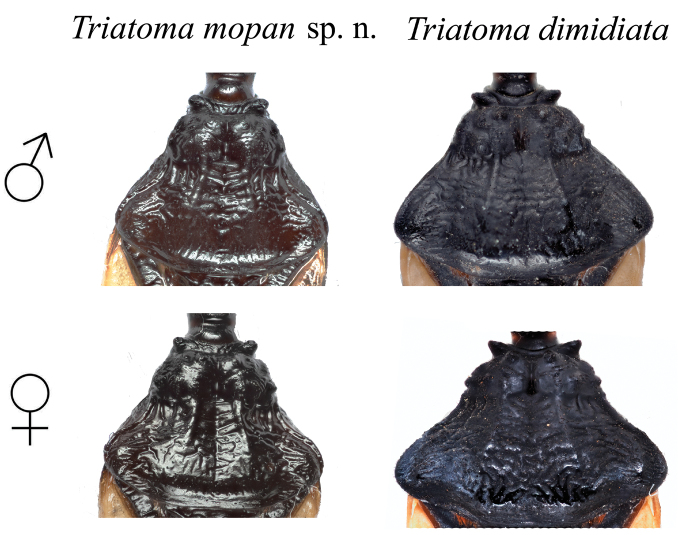
Comparison between the pronotum morphology of *T. mopan* and *T. dimidiata* from Jutiapa, Guatemala males and females. Photographs: Silvia Justi.

*Neck* dark, with 1+1 lateral light brown to yellowish spots. Pronotum uniformly dark brown to black, with a distinctive depression forming a straight latitudinal line from the neck to the posterior portion of the pronotum (Figure 8). Anterior lobe rugose, without distinctive tubercles (Figures 3 and 8). Anterolateral angles, short, rounded, in some specimens subconical. Scutellum shallowly rugose, with central median depression heart shaped, apical process about as long as body of scutellum, subcylindrical, slightly downwardly bent at apex. Hemelytra usually surpassing the apex of abdomen but leaving female genital segments exposed. Basal portion of clavus dark brown, apex pale yellow. Corium pale yellow, with the extreme apex black, and with a dark central spot of variable size. Membrane almost as pale as the corium. Legs uniformly dark. Forelegs with 1+1 apical small denticles 2 +1 subapical denticles on both males and females. Middle legs with 1+1 apical small denticles 2 +2 asymmetrical subapical denticles on males and 2 +2 asymmetrical subapical denticles on females (Figure 4).

**Figure 9.**
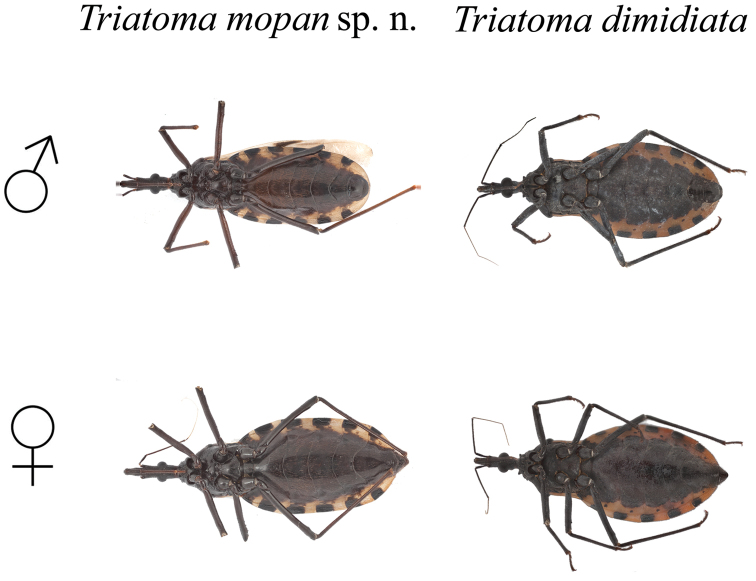
Comparison between ventral views of from *T. mopan* and *T. dimidiata* from Jutiapa, Guatemala males and females. Photographs: Silvia Justi and Raquel Lima.

*Abdomen* ventrally convex, minutely striate transversally, with scarce yellow pilosity. Mostly dark brown, with yellow spots extending from the connexival suture (Figure 9). Spiracles close or adjacent to connexival suture, usually surrounded by a spot slightly darker than tegument (Figure 9). Connexival segments piceous or black on anterior third to half across entire width, almost always as an extension of the piceous portion of the abdomen, pale posteriorly.

*External genitalia* dark brown to black, almost round, with setae darker than the rest of the tegument in males; triangular, with long reddish setae on females (Figure 9).

####### Distribution.

To date, the species is only known from the type locality.

####### Type-locality.

Rio Frio cave, Cayo District, Belize, coordinates 16.956939/-88.979675.

####### Etymology.

The specific epithet *mopan* was chosen to honor the indigenous Mopan people, one of the Mayan groups historically and presently in this area of Belize and Guatemala, and comprises the lineage previously referred to as T. sp. aff. dimidiata (Group 4 – cave; Monteiro et al. 2013, Stevens et al. 2014, Dorn et al. 2016, Justi et al. 2018).

####### Host-parasite data.

Specimens of *T. mopan* sp. n. collected prior to its description, in the same Rio Frio cave, were found to be infected with *Trypanosoma cruzi* (Monroy, unpublished data). The type series was not investigated for parasite infection in order to preserve the integrity of the samples.

Earlier studies completed by our research group, identifying blood sources on specimens of the then undescribed *T. mopan* collected in the same Rio Frio cave indicate that this species feeds on human, pig, goat or sheep, rat, mouse, duck, bat, opossum, and synanthropic and wild vertebrates (Stevens et al. 2014).

####### Bionomics.

Found in caves, in cracks in rocks near water, in humid environment.

## Results

### Morphological and morphometric study

**Figure 10.**
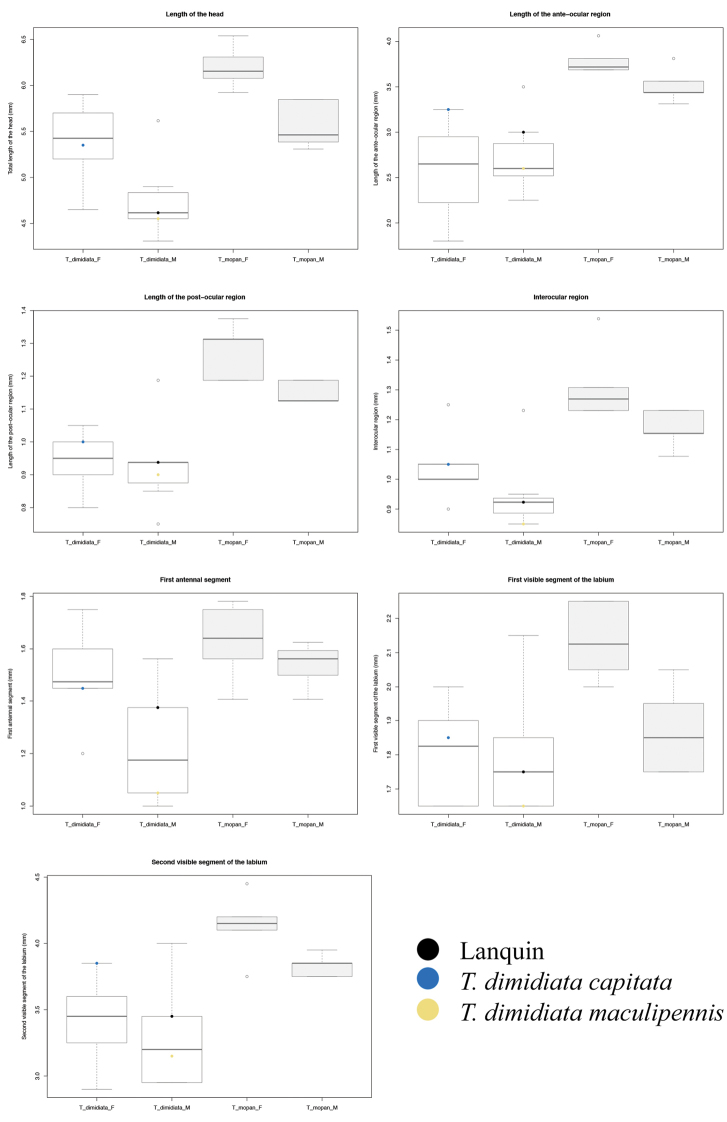
Barplot of the character measures that were significantly different for at least one comparison between male and females of *T. mopan* and *T. dimidiata* (see Table 3).

Character measures used for the description were compared separately for males and females between *T. mopan* and the morphotypes of *T. dimidiata* (Table 3). Barplots showing the mean and standard deviation for each of the significantly different characters are shown on Figure 10, and the bar plots for all the characters are shown in the Suppl. material 1. After the correction for multiple comparisons five of the 18 characters varied between species for females and six of 17 for males (because of missing third and fourth antennae segments, not all 19 characters were measured for *T. dimidiata* (see Table 3). Four characters were significantly different between species for both females and males (HL, POR, R3 and SYN. For females, the two species also differed for the R1 and R2. For males, A1 and AOR differed between species (Table 3).

**Table 3.** Character measurements (mm) of *T. mopan* and *T. dimidiata* specimens and significance of t-test results for the comparison between the sexes of both species. Key: asterisk (*) significant value based on Benjamini-Hochberg multiple comparison False Discovery Rate, FDR = 0.05. n.s. non-significant value, N/A – not available.

Character	*T. mopan* females			*T. dimidiata* females				*T. mopan* males			*T. dimidiata* males			
	N	Mean (mm)	Min–Max (mm)	N	Mean (mm)	Range (mm)	P-value	N	Mean (mm)	Range (mm)	N	Mean (mm)	Range (mm)	P-value
A1	6	1.63	1.406–1.781	6	1.492	1.200–1.750	n.s.	5	1.538	1.406–1.625	6	1.223	1.000–1.563	< 0.02*
A2	6	4.719	4.500–5.281	5	4.2	3.400–4.800	n.s.	5	4.769	4.125–5.313	4	4.255	4.000–4.719	n.s.
A3	3	4.365	4.219–4.531	3	3.567	3.100–3.800	n.s.	5	4.194	3.688–4.469	0	N/A	N/A	N/A
A4	1	1.844	1.844–1.844	3	2.617	1.750–3.100	N/A	5	2.725	0.000–4.188	0	N/A	N/A	N/A
AOR	6	3.781	3.688–4.063	3	2.567	1.800–3.250	n.s.	5	3.513	3.313–3.813	7	2.734	2.250–3.500	< 0.002*
BOS	6	1.823	1.563–2.063	3	1.717	1.550–1.950	n.s.	5	1.525	1.188–2.000	7	1.857	1.500–2.063	n.s.
HL	6	6.192	5.923–6.538	6	5.383	4.650–5.900	< 0.005*	5	5.569	5.308–5.846	7	4.758	4.308–5.615	< 0.005*
LOP	6	5.295	5.000–6.000	6	4.767	3.950–5.400	n.s.	5	4.723	4.308–5.077	7	4.805	3.950–7.000	n.s.
POR	6	1.281	1.188–1.375	6	0.942	0.800–1.050	< 0.0001*	5	1.15	1.125–1.188	7	0.929	0.750–1.188	< 0.005*
POS	6	1.458	1.000–1.688	3	1.55	1.200–1.750	n.s.	5	1.425	0.813–1.625	7	1.307	1.063–1.625	n.s.
R1	6	2.133	2.000–2.250	6	1.808	1.650–2.000	< 0.002	5	1.87	1.750–2.050	6	1.8	1.650–2.150	n.s.
R2	6	4.133	3.750–4.450	6	3.417	2.900–3.850	< 0.002	5	3.83	3.750–3.950	6	3.292	2.950–4.000	n.s.
R3	6	1.202	1.000–1.610	6	0.967	0.850–1.050	< 0.05 n.s.	5	1.2	1.150–1.250	6	0.933	0.700–1.150	< 0.01*
S	6	1.308	1.231–1.538	6	1.033	0.900–1.250	< 0.005*	5	1.169	1.077–1.231	7	0.95	0.850–1.231	< 0.005*
TL	6	34.58	32.170–38.000	6	33.12	28.830–34.200	n.s.	5	28.73	26.670–30.170	7	29.98	23.830–35.500	n.s.
TS	6	3.281	2.750–3.500	3	3.15	2.500–3.650	n.s.	5	2.95	2.750–3.188	7	3.164	2.750–3.688	n.s.
WAE	6	2.487	2.385–2.692	6	2.492	2.200–2.800	n.s.	5	2.323	2.231–2.462	7	2.415	2.154–2.900	n.s.
WE	6	0.599	0.500–0.625	6	0.733	0.600–0.900	n.s.	5	0.594	0.563–0.625	7	0.654	0.500–0.800	n.s.
WOP	6	6.526	6.077–7.385	6	6.633	2.800–8.000	n.s.	5	6.308	6.231–6.385	7	5.352	3.100–7.385	n.s.

**Table 4.** *Triatoma mopan* character measures for males, females and the holotype.

	Males (mm)				Females (mm)				Holotype (mm)
	Min.	Mean	Max.	sd	Min.	Mean	Max.	sd	
A1	1.41	1.538	1.63	0.086	1.41	1.63	1.78	0.138	1.59
A2	4.13	4.77	5.31	0.483	4.5	4.718	5.28	0.293	5.19
A3	3.69	4.194	4.47	0.303	4.22	4.363	4.53	NA	4.31
A4	2.75	2.952	3.19	0.197	2.75	3.282	3.5	0.279	3.13
AOR	3.31	3.512	3.81	0.189	3.69	3.782	4.06	0.145	3.56
BOS	1.19	1.526	2	-0.332	1.56	1.823	2.06	0.191	1.5
HL	5.31	5.57	5.85	0.261	5.92	6.193	6.54	0.217	5.85
LOP	4.31	4.724	5.08	0.305	5	5.295	6	0.371	4.77
POR	1.13	1.154	1.19	0.033	1.19	1.282	1.38	0.076	1.19
POS	0.81	1.426	1.63	-0.349	1	1.46	1.69	0.28	1.63
R1	1.75	1.87	2.05	0.130	2	2.133	2.25	0.103	2.05
R2	3.75	3.83	3.95	0.084	3.75	4.133	4.45	0.225	3.85
R3	1.15	1.2	1.25	0.05	1	1.202	1.61	0.211	1.25
S	1.08	1.168	1.23	0.063	1.23	1.308	1.54	0.120	1.15
TL	26.67	28.74	30.17	1.677	32.17	34.58	38	2.056	29.67
TS	2.84	3.408	4.19	NA	1.84	1.84	1.84	NA	3.13
WAE	2.23	2.324	2.46	0.084	2.38	2.485	2.69	0.117	2.31
WE	0.56	0.592	0.63	0.025	0.5	0.602	0.63	0.052	0.59
WOP	6.23	6.306	6.38	0.075	6.08	6.525	7.38	0.458	6.38

**Figure 11.**
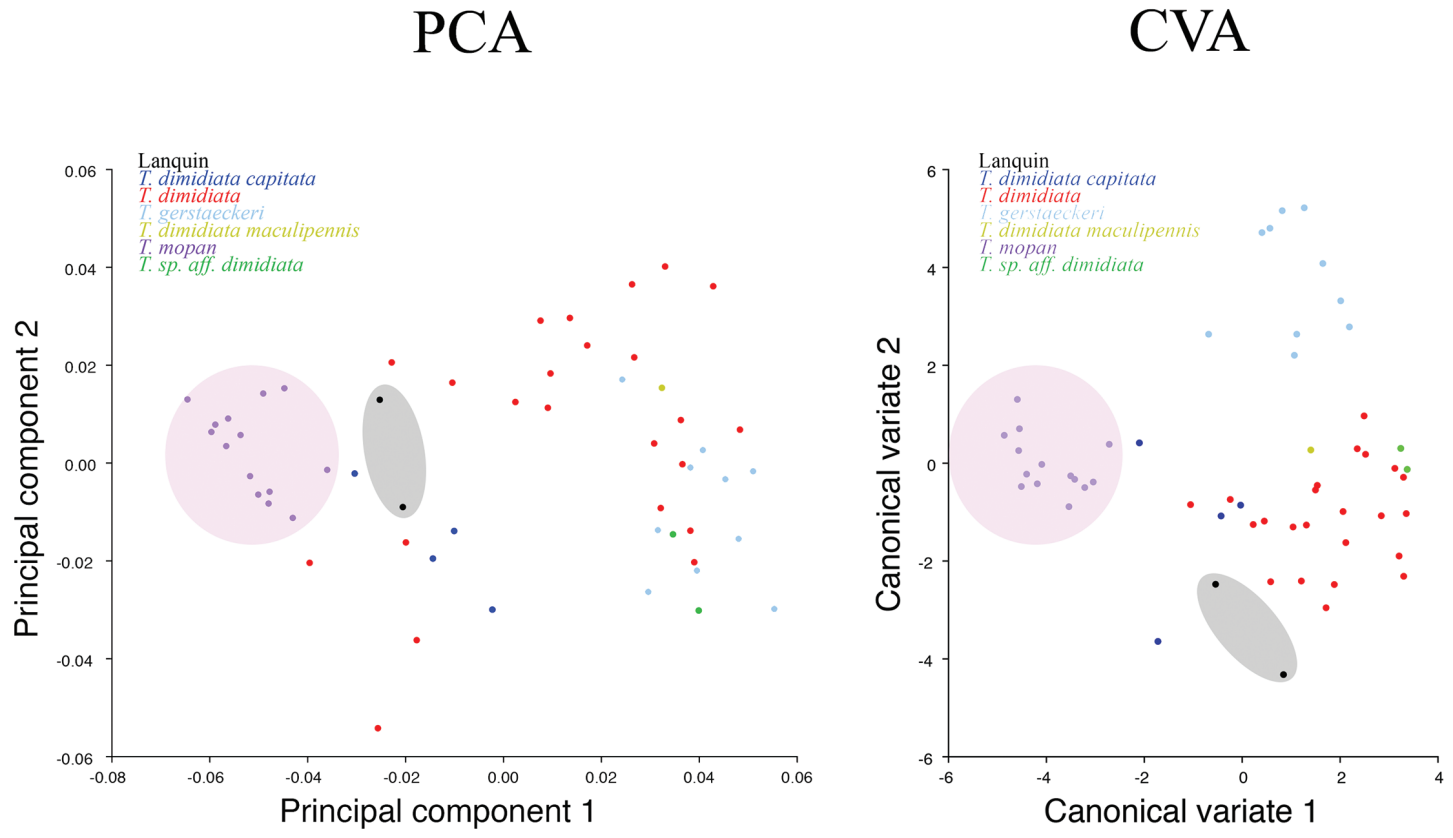
PCA and CVA of the Morphometric comparison of the heads of *T. mopan*, *T. gerstaeckeri*, *Triatoma* from Lanquin and *T. dimidiata* different morphotypes. Note that both *T. mopan* and *Triatoma* from Lanquin form clusters separated from the other species.

**Figure 12.**
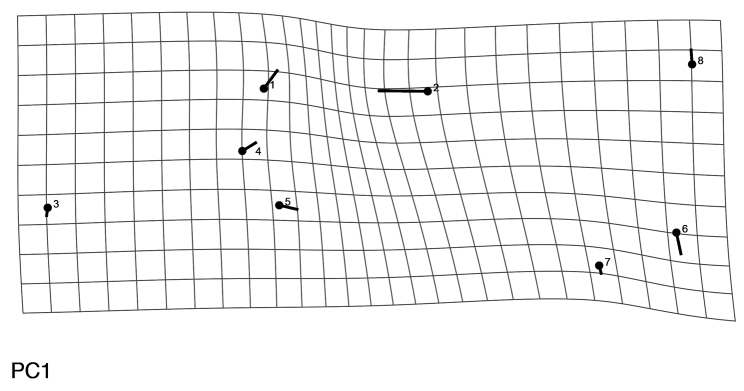
PCA thin plate showing that landmarks 1 and 2 are the most variable within the compared specimens.

Head shape comparison between *T. mopan* specimens and *T. dimidiata* and *T. gerstaeckeri* revealed a unique separate cluster comprising *T. mopan* for both PCA and CVA (Figure 11). ANOVA results led to the rejection of the null hypothesis (p<0.0001), that is, our results indicate that the variance between the means of the groups are different. PCA results show that most of the variation is from landmarks corresponding to the antennal tubercle and the anterior portion of the eye (Figure 12).

### Phylogenetic reconstruction and genetic distances

**Figure 13.**
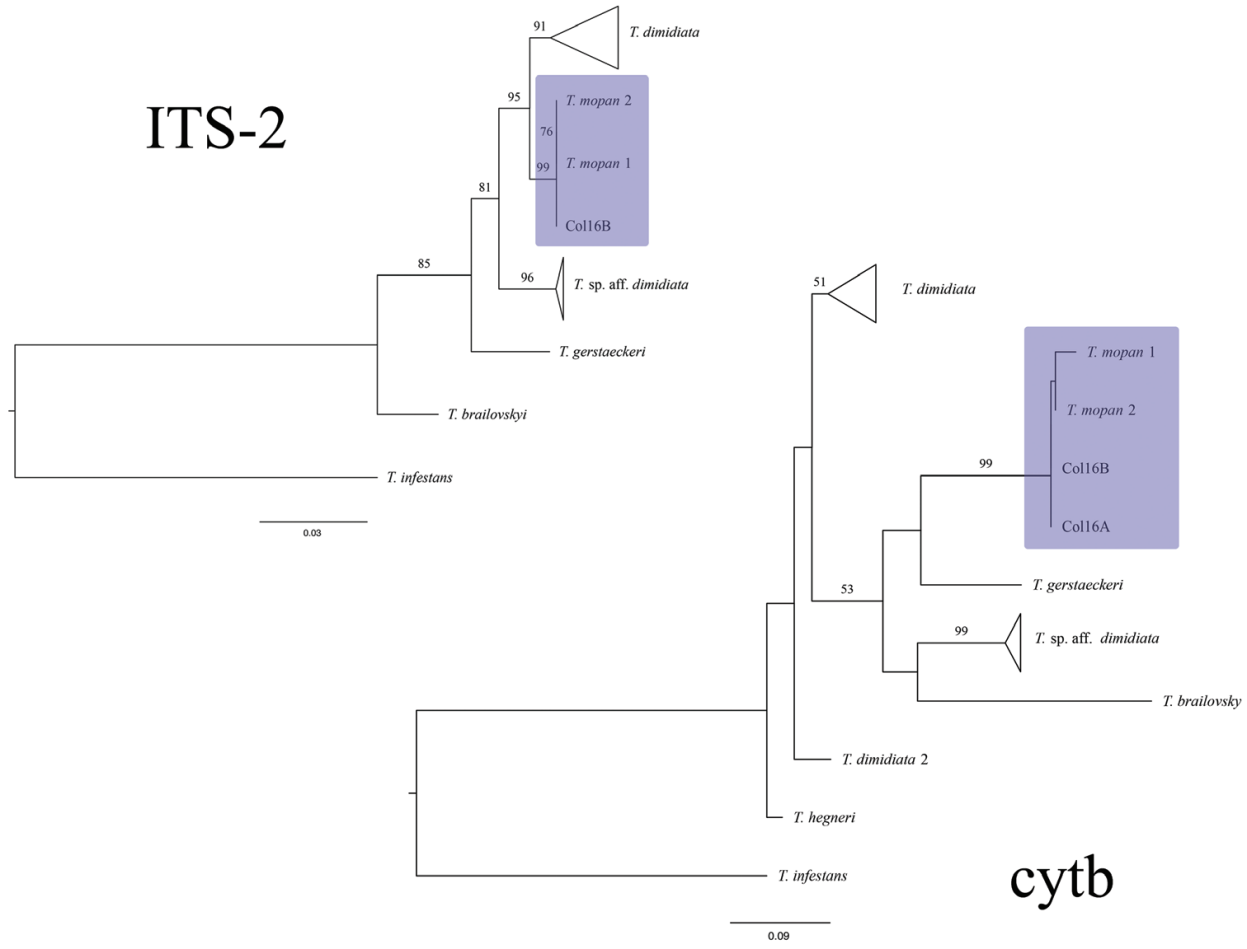
ML phylogenies reconstructed using ITS-2 (right) and cytb (left) sequences. Numbers above branches represent bootstrap support > 50. *T. mopan* clade is highlighted in purple.

Both ML phylogenies recovered *T. mopan* as an independently evolving lineage, with the highest bootstrap support recovered for each phylogeny. *Triatoma mopan* was recovered as a sister taxon to *T. dimidiata* upon ITS-2 phylogenetic reconstruction and as sister to *T. gerstaeckeri* when cytb was used for the reconstruction (Figure 13).

**Figure 14.**
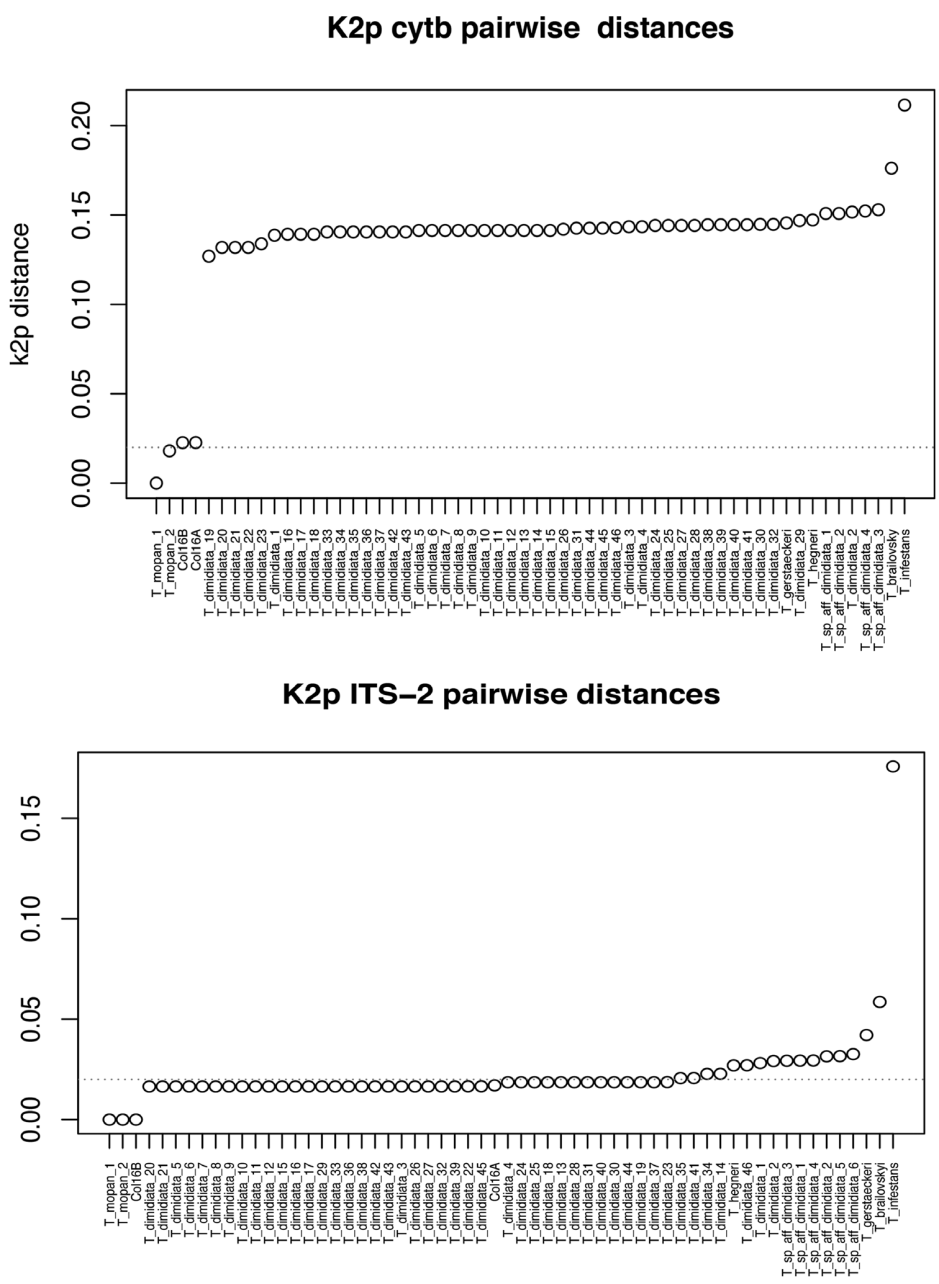
Plot of the K2-p distances calculated pairwise between *T. mopan* and all the other specimens used for the phylogenetic reconstruction. Dotted line indicates 2% divergence.

Pairwise Kimura 2-parameter genetic distances revealed *T. mopan* to diverge at least 2% from the other lineages when distances were calculated for ITS-2; while for cytb the minimum pairwise distance between *T. mopan* and the closest examined species increases to about 10% (Figure 14).

## Discussion

Phylogenetic studies of the diverse *T. dimidiata* have long shown the taxa to be composed of at least three independently evolving lineages (Dorn et al. 2007, 2016, Bargues et al. 2008, Herrera-Aguilar et al. 2009, Monteiro et al. 2013, Justi et al. 2018). One such lineage, the *Triatoma* from the Rio Frio cave in Belize, was first observed to be a separate phylogenetic species by Monteiro et al. 2013, and referred to as *T. dimidiata* group 4. Later, more comprehensive studies confirmed the specific phylogenetic status of the lineage referred to as T. sp. aff. dimidiata – cave, pending the formal description of the new species (Dorn et al. 2016, Justi et al. 2018). In this study, we describe the *Triatoma* lineage from the Rio Frio cave, in Belize and and name it *Triatoma mopan*, after the Mopan people of that area.

We have compared *T. mopan* with *T. dimidiata* using different systematic approaches: classic morphology, geometric morphometric and molecular phylogeny, and the results agree in that these are separate species. Diagnostic characters were observed on the pronotum, legs and abdomen and PCA and CVA results place both in separate clusters (Figures 3, 4, 5, 11).

**Figure 15.**
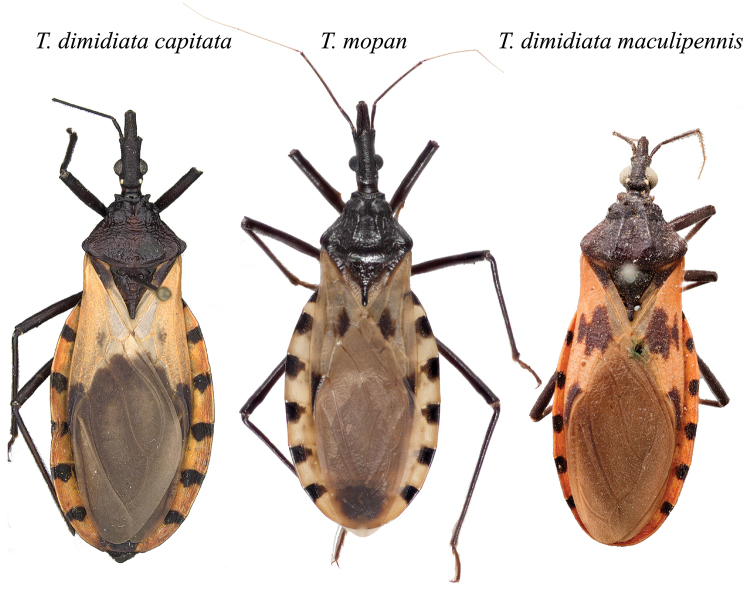
*Triatoma dimidiata capitata* (Photo: Rachel Diaz-Bastin, California Academy of Sciences), *T. mopan* (Photo: Raquel Lima-Cordón), and *T. dimidiata maculipennis* (Photo: © Schurian / MfN.berlin) holotypes. Photographs are not to scale.

The morphological comparison of *T. mopan* with the description of *T. dimidiata* (Lent and Wygodzinsky 1979) and photos of the holotypes of *T. dimidiata capitata* and *T. dimidiata maculipennis* (Figure 15), along with the molecular phylogenetic results published previously (Dorn et al. 2007, 2009, 2016, Bargues et al. 2008, Monteiro et al. 2013, Justi et al. 2018) show the uniqueness of the Rio Frio species. All diagnostic characters and the description of the species, allowed us to observe a combination of characters unique to *T. mopan*.

**Figure 16.**
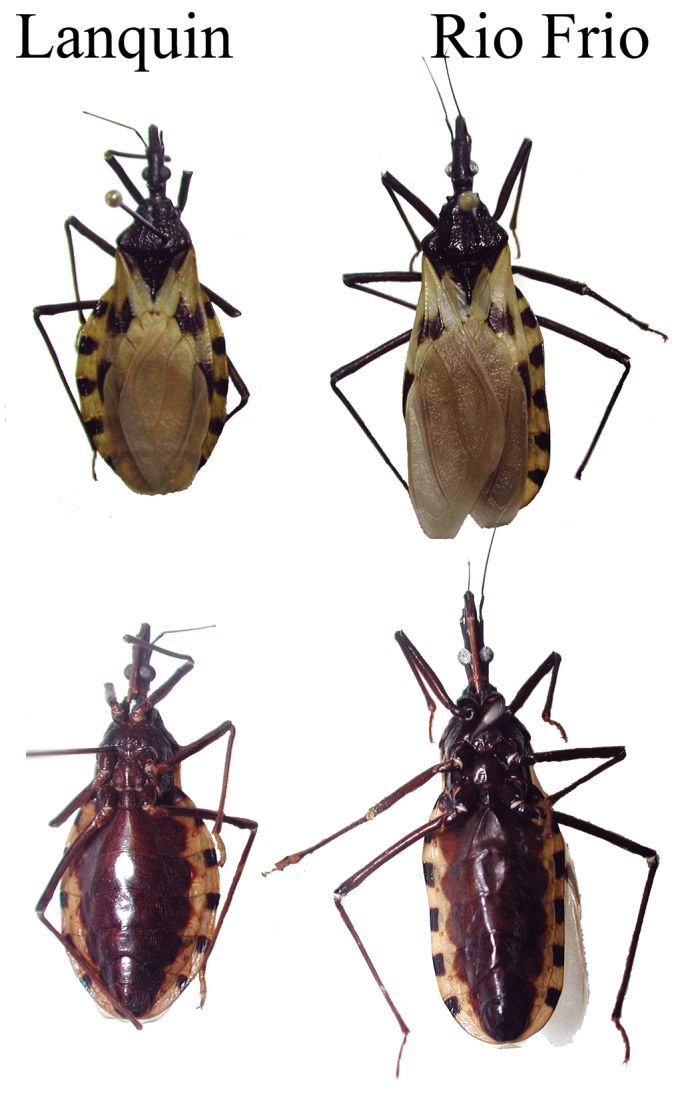
Comparison between *Triatoma* from Lanquin and *T. mopan*. Photographs to scale. Photo: Carolina Dale.

Lent and Wygodzinsky (1979) observed 160 specimens of *T. dimidiata* and concluded that the morphological variation observed within the taxa displays a clinal pattern with size increasing southwards. Amongst those, they describe five specimens from the Lanquin cave, in Guatemala, highlighting differences in the length and ratio of the antennae, and mentioning characteristics related to the cave environment, such as diminished pigmentation and eye and ocelli size when compared with non-cave populations of *T. dimidiata*. The authors also stated that the single specimen examined from the Rio Frio cave “*is identical phenotypically with the specimens from Lanquin cave*”. In light of this statement, we compared *T. mopan* with the Lent and Wygodzinsky (1979) description and to two specimens from the Lanquin cave population and noticed distinctive characteristics in the head (Table 5), wing, and abdomen coloration pattern (Figure 16) that clearly separate *T. mopan* and the Lanquin population of *Triatoma*. Likely convergent evolution due to the cave environment is observed in the diminished pigmentation and relative smaller eye and ocelli sizes and the absence of tubercles on the anterior lobe of the pronotum. Even though Lent and Wygodzinsky stated that these are *T. dimidiata* populations from caves, it is not possible to taxonomically place these Lanquin and Rio Frio cave specimens using their key to the species of *Triatoma* (combination of characters described on dychotomy 39 does not correspond to either population).

**Table 5.** Character comparison between *T. mopan*, *T. dimidiata*, and *Triatoma* from Lanquin cave, based on the description of Lent and Wygodzinsky (1979) for the latter.

	*T. mopan*	*Triatoma* Lanquin	*T. dimidiata*
**Antenna**	first antennal segment attaining to the level of or surpassing the apex of clypeus	first antennal segment surpassing the apex of clypeus	first antennal segment attaining to the level of the apex of clypeus
**POR/AOR**	2.8–3.2	4	2.5–3
**Ratio antennae segments**	1:2.7–3.4:2.5-2.6:1.15	1:2.5:2.2:2.3	1:3.2–3.8:2.5:2.2.
**Eyes**	surpassing the level of the ventral surface of the head	not surpassing the level of the ventral surface of the head	attaining to the level of the ventral surface of the head

The geometric morphometric comparison of the head shape (PCA and CVA) also placed the Lanquin specimens closer to *T. dimidiata* (but separate from) than to *T. mopan*, which forms a unique separate cluster (Figure 11). Similar results were previously found, placing the Lanquin population in a separate cluster from the *T. dimidiata* populations (Bustamante et al. 2004). Additionally, previous molecular phylogeny results have shown that the *Triatoma* specimens collected in the Lanquin cave fall within *T. dimidiata* s.s (Monteiro et al. 2013, Dorn et al. 2016), while the ones collected in the Rio Frio cave comprise an independently evolving lineage. These results were confirmed by the broader phylogenetic recontruction and comparison of genetic distances performed for this study (Figures 13, 14). The widely distinct morphology of the Lanquin cave specimens, combined with these phylogenetic results show the need for a deeper study of this population to better understand its evolution and taxonomy.

The comparison of the morphology of *T. mopan* with *T. dimidiata* shows a clear trend in cave adaptation evolution. Besides the diminished overal pigmentation of the specimens, *T. mopan* exhibits much denser sensillae on the visible labial segments (Figure 7), which are significantly longer than in *T. dimidiata*, probably to compensate for the reduced visual cues in such an environment.

In combination, the morphological characters with molecular phylogeney and geometric morphometry of the head show that *T. mopan* is an independently evolving lineage, separate from *T. dimidiata*. The comparison with the types of *T. dimidiata maculipennis*, *T. dimidiata capitata* and the description given by Lent and Wygodzinsky (1979) for *T. dimidiata*, in the absence of the type, make it clear that *T. mopan* is a separate species, not previously formally described as any morphotype or subspecies of *T. dimidiata*.

## Supplementary Material

XML Treatment for Triatoma mopan

## Supplementary materials

Supplementary material 1
*T. mopan* × *T. dimidiata* - boxplots comparing the characters. This is an open access article distributed under the terms of the Creative Commons Attribution License (CC BY 4.0), which permits unrestricted use, distribution, and reproduction in any medium, provided the original author and source are credited.Patricia L. Dorn, Silvia A. Justi, Carolina Dale, Lori Stevens, Cleber Galvão, Raquel Lima-Cordón, Carlota Monroy

Supplementary material 2
*T. mopan* description - R codes. This is an open access article distributed under the terms of the Creative Commons Attribution License (CC BY 4.0), which permits unrestricted use, distribution, and reproduction in any medium, provided the original author and source are credited.Patricia L. Dorn, Silvia A. Justi, Carolina Dale, Lori Stevens, Cleber Galvão, Raquel Lima-Cordón, Carlota Monroy
