# Supplementary material for: Description of Triatoma mopan sp. n. from a cave in Belize (Hemiptera, Reduviidae, Triatominae)
Source: Zookeys. 2018 Jul 17;(775):69–95. doi: 10.3897/zookeys.775.22553 (PMC6058004; doi:10.3897/zookeys.775.22553)
Supplement: Supplementary material 1 — T. mopan × T. dimidiata - boxplots comparing the characters. [file zookeys-775-069-s001.pdf]

T. mopan X T. dimidiata

Silvia Justi

2/26/2018

```
data<-read.csv("measures_mopan.csv", header=T)
Lanquin<-data[3, ]
Capitata <- data[20,]
Macullipennis <-data[21,]
T_dim_F<-subset(data, Species=='T_dimidiata_F')
T_mop_F<-subset(data, Species=='T_mopan_F')
T_dim_M<-subset(data, Species=='T_dimidiata_M')
T_mop_M<-subset(data, Species=='T_mopan_M')
```

```
boxplot(data$TL ~ data$Species, ylab ="Total length of the body (mm)",
        border= c("gray45","gray45","gray45","gray45"),
        col=c("white", "white", "gray95", "gray95" ),
        main =  "Comparison of total length of the body")
stripchart(Lanquin$TL, vertical=TRUE, add=TRUE,
           method="stack", col='black', pch=19, at = 2)
stripchart(29, vertical=TRUE, add=TRUE,
           method="stack", col='red', pch=19, at = 2)
stripchart(33, vertical=TRUE, add=TRUE,
           method="stack", col='red', pch=19, at = 1)
stripchart(Capitata$TL, vertical=TRUE, add=TRUE,
           method="stack", col='dodgerblue3', pch=19, at = 1)
stripchart(Macullipennis$TL, vertical=TRUE, add=TRUE,
           method="stack", col='lightgoldenrod', pch=19, at = 2)
legend("topright", legend = c("Lanquin L&W","Lanquin", "T. dimdiata capitata",
                              "T. dimdiata maculipennis"),
       col = c('red','black','dodgerblue3','lightgoldenrod'),
cex = 0.5, pch = 19, bty = "n")
```

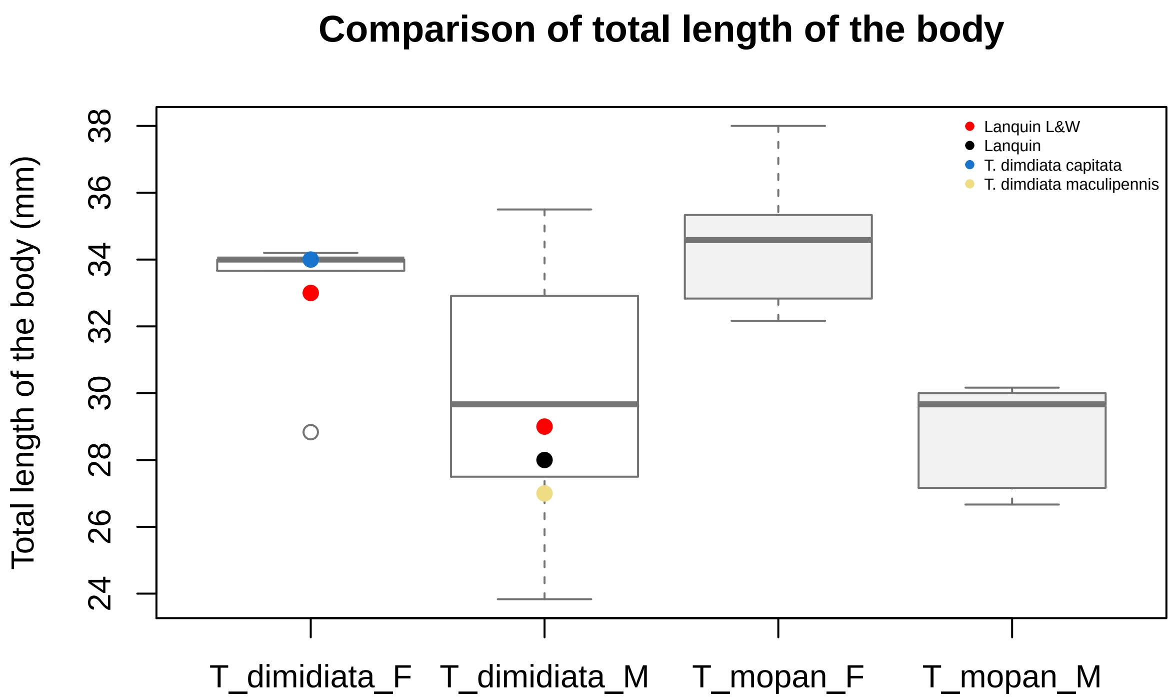


```r
boxplot(data$WOP  ~ data$Specie, ylab ="Width of the pronotum (mm)",
         border= c("gray45","gray45","gray45","gray45"),
        col=c("white", "white", "gray95", "gray95" ),
        main =  "Comparison of width of the pronotum ")
stripchart(Lanquin$WOP, vertical=TRUE, add=TRUE,
           method="stack", col='black', pch=19, at = 2)
stripchart(Capitata$WOP, vertical=TRUE, add=TRUE,
           method="stack", col='dodgerblue3', pch=19, at = 1)
stripchart(Macullipennis$WOP, vertical=TRUE, add=TRUE,
           method="stack", col='lightgoldenrod', pch=19, at = 2)
legend("topright", legend = c("Lanquin", "T. dimdiata capitata",
                              "T. dimdiata maculipennis"),
       col = c('black','dodgerblue3','lightgoldenrod'),
cex = 0.5, pch = 19, bty = "n")
```

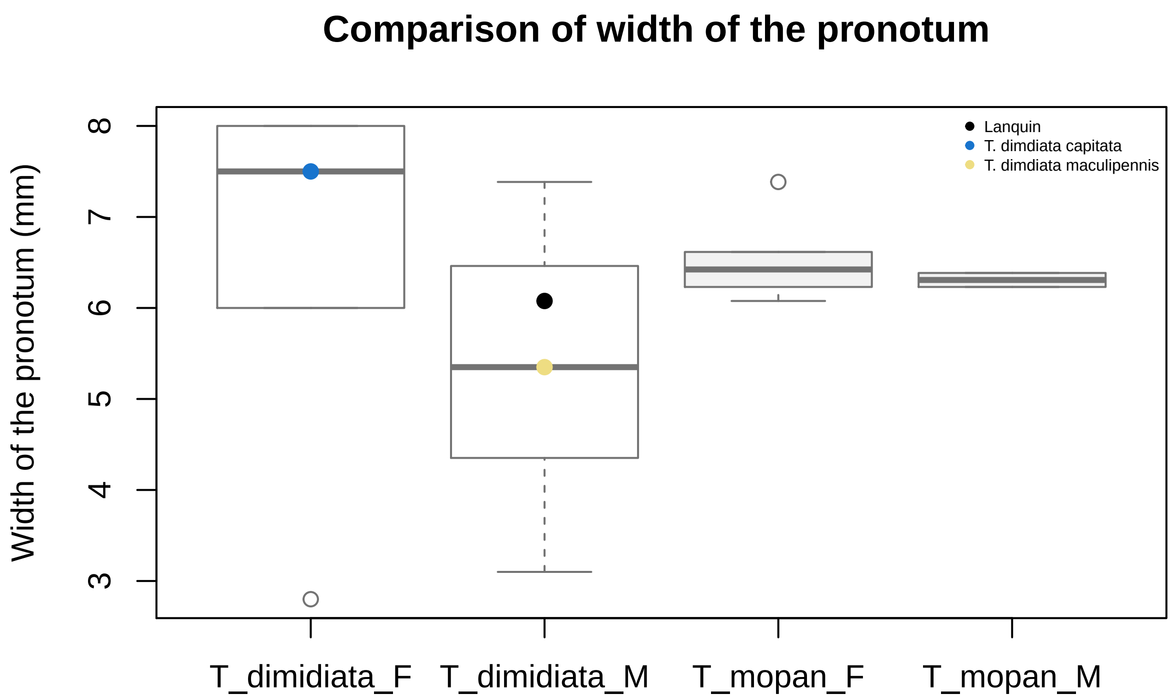


```r
boxplot(data$HL ~ data$Specie, ylab ="Total length of the head (mm)",
        border= c("gray45","gray45","gray45","gray45"),
        col=c("white", "white", "gray95", "gray95" ),
        main =  "Comparison of length of the head ")
stripchart(Lanquin$HL, vertical=TRUE, add=TRUE,
           method="stack", col='black', pch=19, at = 2)
stripchart(Capitata$HL, vertical=TRUE, add=TRUE,
           method="stack", col='dodgerblue3', pch=19, at = 1)
stripchart(Macullipennis$HL, vertical=TRUE, add=TRUE,
           method="stack", col='lightgoldenrod', pch=19, at = 2)
legend("topright", legend = c("Lanquin", "T. dimdiata capitata",
                              "T. dimdiata maculipennis"),
       col = c('black','dodgerblue3','lightgoldenrod'),
cex = 0.5, pch = 19, bty = "n")
```

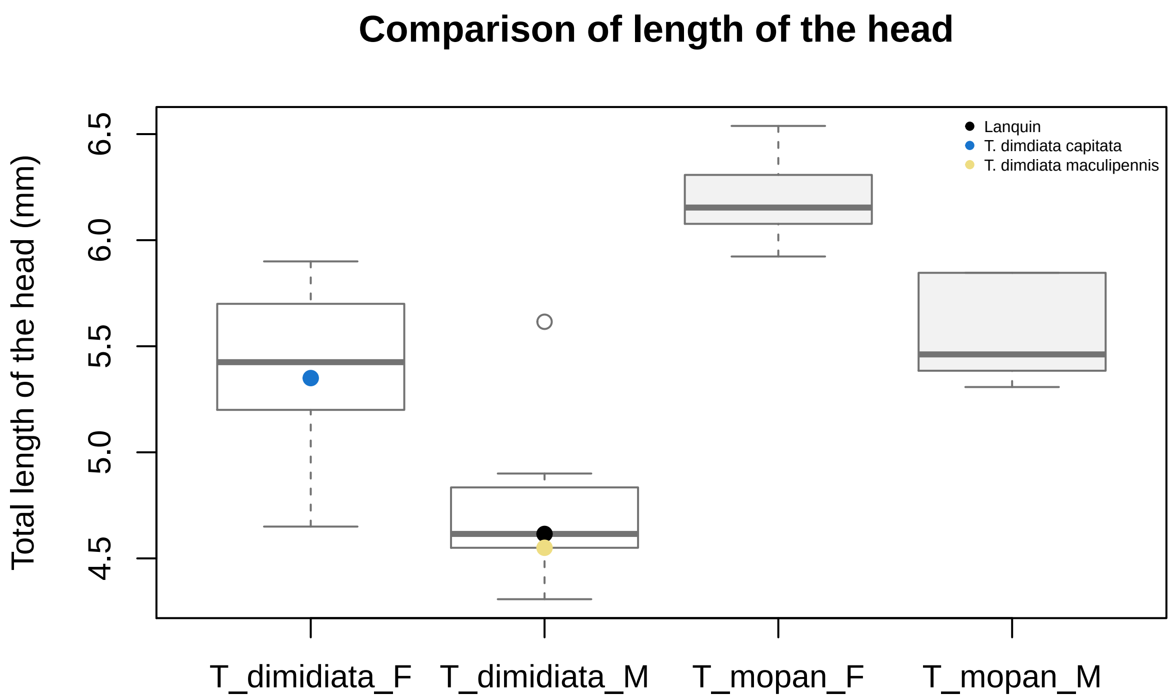


```
boxplot(data$WAE ~ data$Specie, ylab ="Width across eyes(mm)",
         border= c("gray45","gray45","gray45","gray45"),
        col=c("white", "white", "gray95", "gray95" ),
        main =  "Comparison of width across eyes")
stripchart(Lanquin$WAE, vertical=TRUE, add=TRUE,
           method="stack", col='black', pch=19, at = 2)
stripchart(Capitata$WAE, vertical=TRUE, add=TRUE,
           method="stack", col='dodgerblue3', pch=19, at = 1)
stripchart(Macullipennis$WAE, vertical=TRUE, add=TRUE,
           method="stack", col='lightgoldenrod', pch=19, at = 2)
legend("topright", legend = c("Lanquin", "T. dimdiata capitata",
                              "T. dimdiata maculipennis"),
       col = c('black','dodgerblue3','lightgoldenrod'),
cex = 0.5, pch = 19, bty = "n")
```

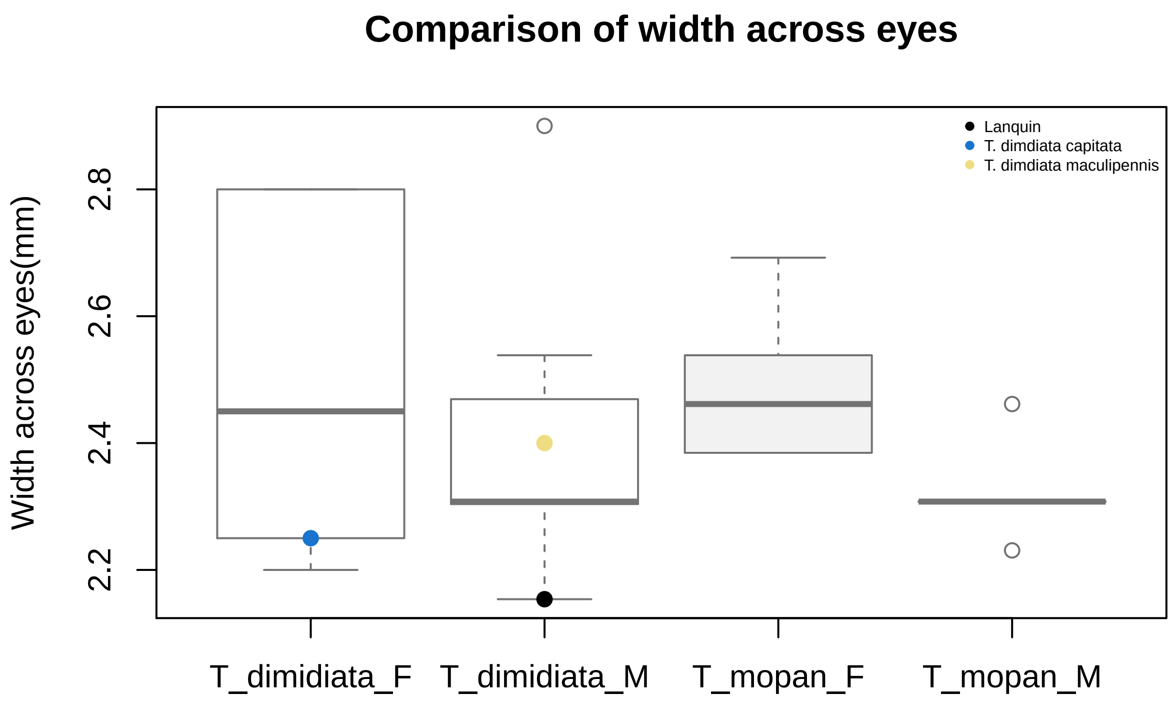


```
boxplot(data$LOP ~ data$Species, ylab ="Total length of the pronotum (mm)",
        border= c("gray45","gray45","gray45","gray45"),
        col=c("white", "white", "gray95", "gray95" ),
        main =  "Comparison of length of the pronotum ")
stripchart(Lanquin$LOP, vertical=TRUE, add=TRUE,
           method="stack", col='black', pch=19, at = 2)
stripchart(Capitata$LOP, vertical=TRUE, add=TRUE,
           method="stack", col='dodgerblue3', pch=19, at = 1)
stripchart(Macullipennis$LOP, vertical=TRUE, add=TRUE,
           method="stack", col='lightgoldenrod', pch=19, at = 2)
legend("topright", legend = c("Lanquin", "T. dimdiata capitata",
                              "T. dimdiata maculipennis"),
       col = c('black','dodgerblue3','lightgoldenrod'),
cex = 0.5, pch = 19, bty = "n")
```

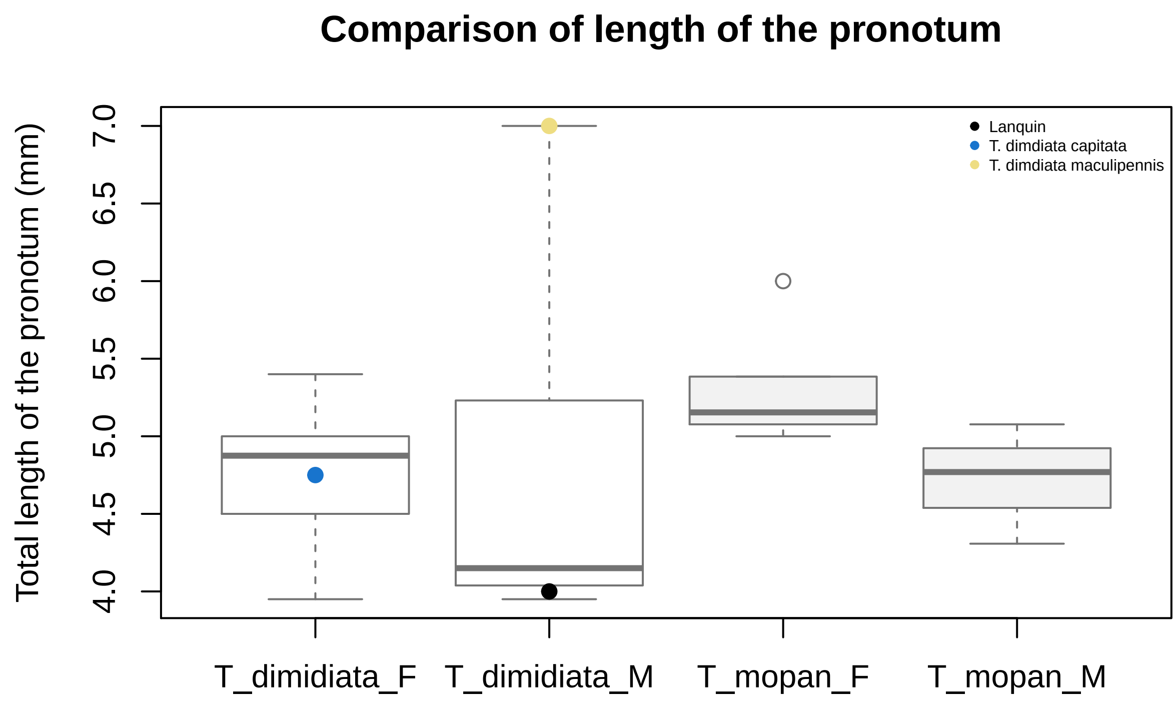


```
boxplot(data$AOR ~ data$Species, ylab ="Length of the ante-ocular region (mm)",
         border= c("gray45","gray45","gray45","gray45"),
        col=c("white", "white", "gray95", "gray95" ),
        main =  "Comparison of length of the ante-ocular region ")
stripchart(Lanquin$AOR, vertical=TRUE, add=TRUE,
           method="stack", col='black', pch=19, at = 2)
stripchart(Capitata$AOR, vertical=TRUE, add=TRUE,
           method="stack", col='dodgerblue3', pch=19, at = 1)
stripchart(Macullipennis$AOR, vertical=TRUE, add=TRUE,
           method="stack", col='lightgoldenrod', pch=19, at = 2)
legend("topright", legend = c("Lanquin", "T. dimdiata capitata",
                              "T. dimdiata maculipennis"),
       col = c('black','dodgerblue3','lightgoldenrod'),
cex = 0.5, pch = 19, bty = "n")
```

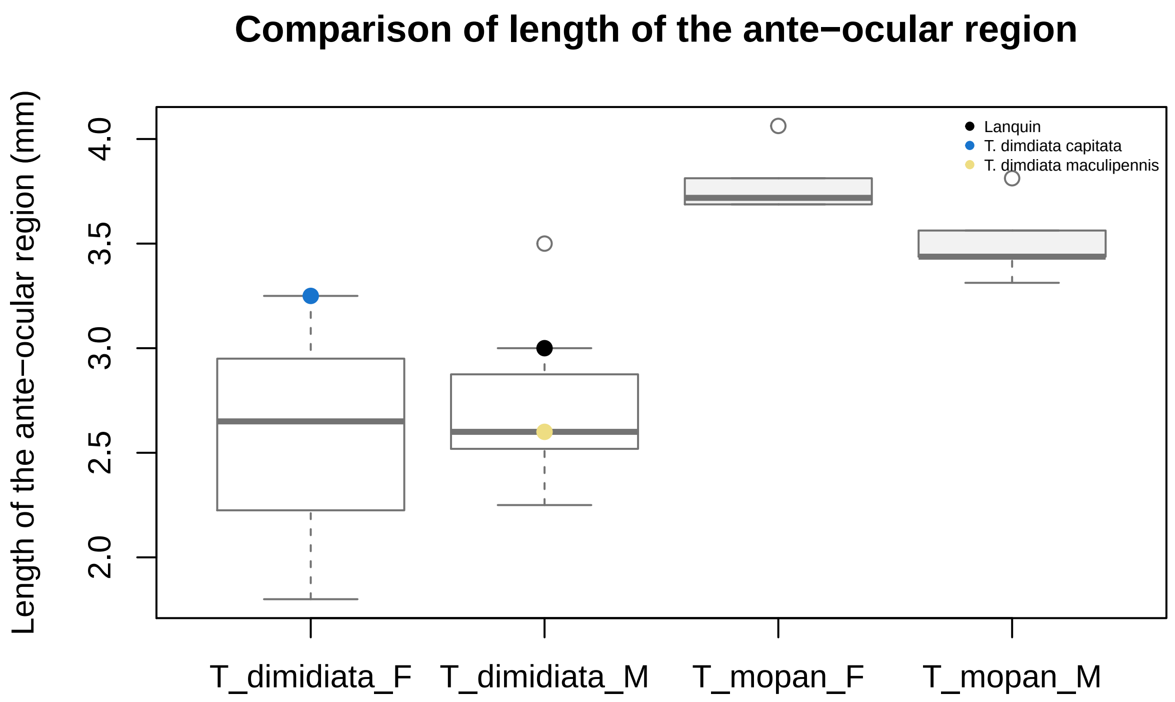


```
boxplot(data$POR~ data$Species, ylab ="Length of the post-ocular region (mm)",
        border= c("gray45","gray45","gray45","gray45"),
        col=c("white", "white", "gray95", "gray95" ),
        main =  "Comparison of length of the post-ocular region ")
stripchart(Lanquin$POR, vertical=TRUE, add=TRUE,
           method="stack", col='black', pch=19, at = 2)
stripchart(Capitata$POR, vertical=TRUE, add=TRUE,
           method="stack", col='dodgerblue3', pch=19, at = 1)
stripchart(Macullipennis$POR, vertical=TRUE, add=TRUE,
           method="stack", col='lightgoldenrod', pch=19, at = 2)
legend("topright", legend = c("Lanquin", "T. dimdiata capitata",
                              "T. dimdiata maculipennis"),
       col = c('black','dodgerblue3','lightgoldenrod'),
cex = 0.5, pch = 19, bty = "n")
```

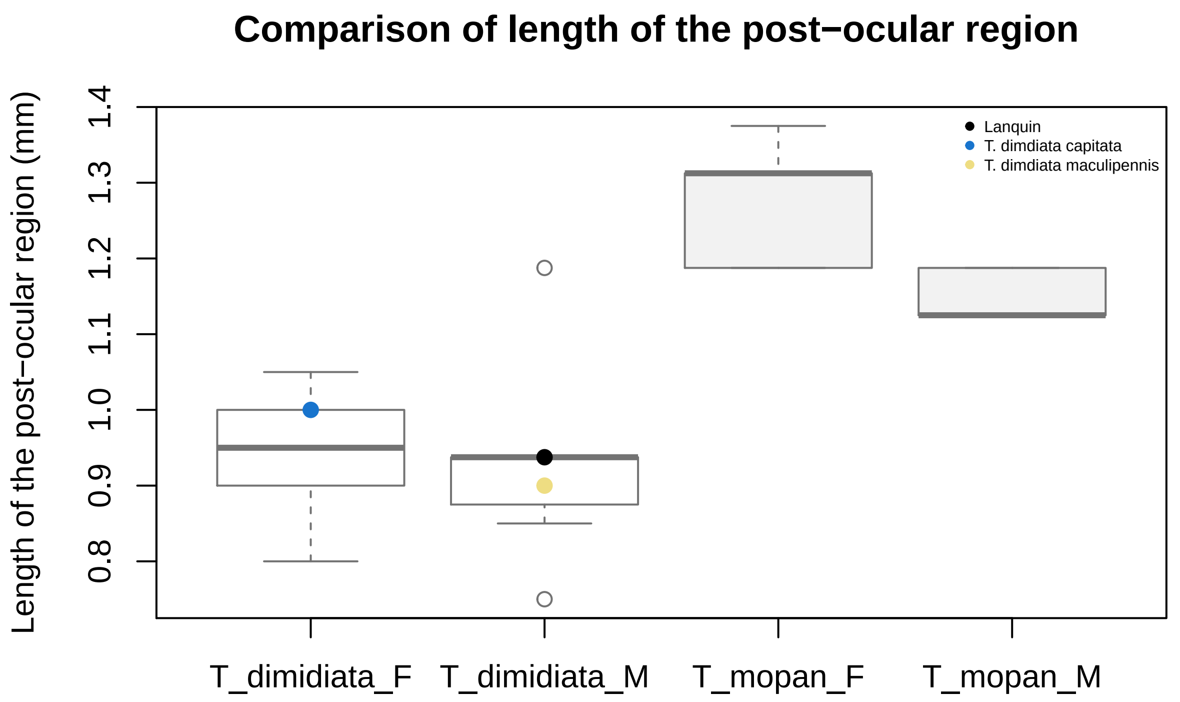


```r
boxplot(data$WE ~ data$Species, ylab ="Width of eyes (mm)",
        border= c("gray45","gray45","gray45","gray45"),
        col=c("white", "white", "gray95", "gray95" ),
        main =  "Comparison of width of eyes ")
stripchart(Lanquin$WE, vertical=TRUE, add=TRUE,
           method="stack", col='black', pch=19, at = 2)
stripchart(Capitata$WE, vertical=TRUE, add=TRUE,
           method="stack", col='dodgerblue3', pch=19, at = 1)
stripchart(Macullipennis$WE, vertical=TRUE, add=TRUE,
           method="stack", col='lightgoldenrod', pch=19, at = 2)
legend("topright", legend = c("Lanquin", "T. dimdiata capitata",
                              "T. dimdiata maculipennis"),
       col = c('black','dodgerblue3','lightgoldenrod'),
cex = 0.5, pch = 19, bty = "n")
```

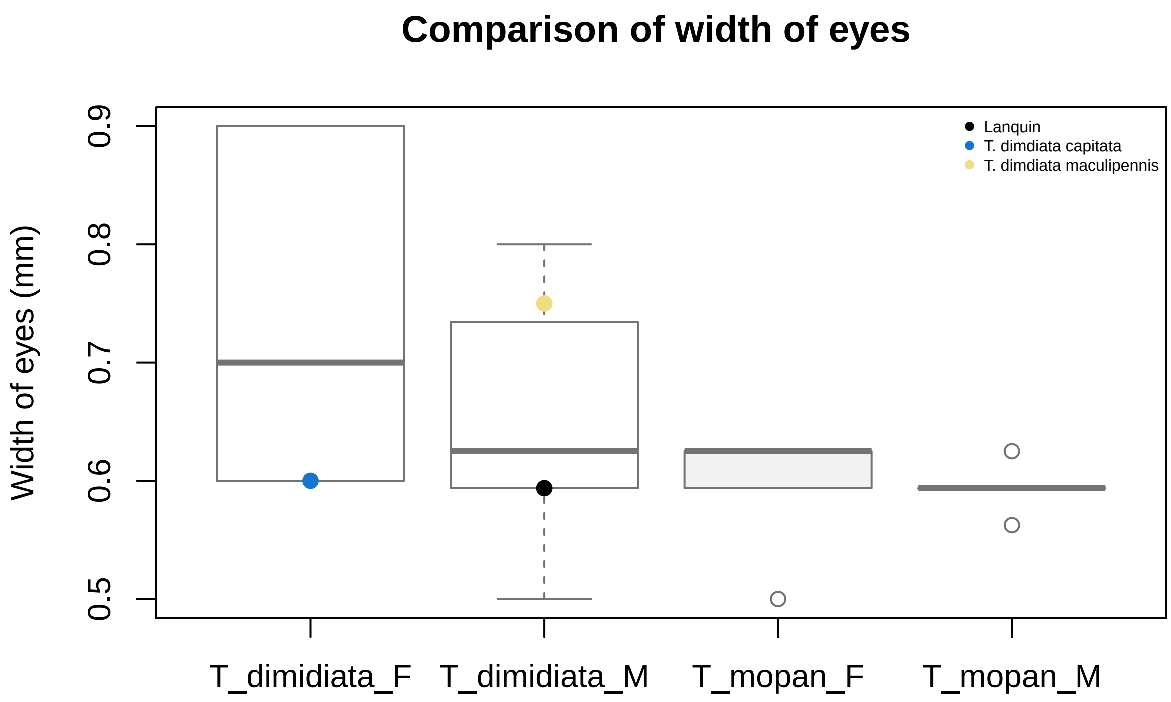


```r
boxplot(data$A1 ~ data$Species, ylab ="First antennal segment (mm)",
        border= c("gray45","gray45","gray45","gray45"),
        col=c("white", "white", "gray95", "gray95" ),
        main =  "First antennal segment")
stripchart(Lanquin$A1, vertical=TRUE, add=TRUE,
           method="stack", col='black', pch=19, at = 2)
stripchart(Capitata$A1, vertical=TRUE, add=TRUE,
           method="stack", col='dodgerblue3', pch=19, at = 1)
stripchart(Macullipennis$A1, vertical=TRUE, add=TRUE,
           method="stack", col='lightgoldenrod', pch=19, at = 2)
legend("topright", legend = c("Lanquin", "T. dimdiata capitata",
                              "T. dimdiata maculipennis"),
       col = c('black','dodgerblue3','lightgoldenrod'),
       cex = 0.5, pch = 19, bty = "n")
```

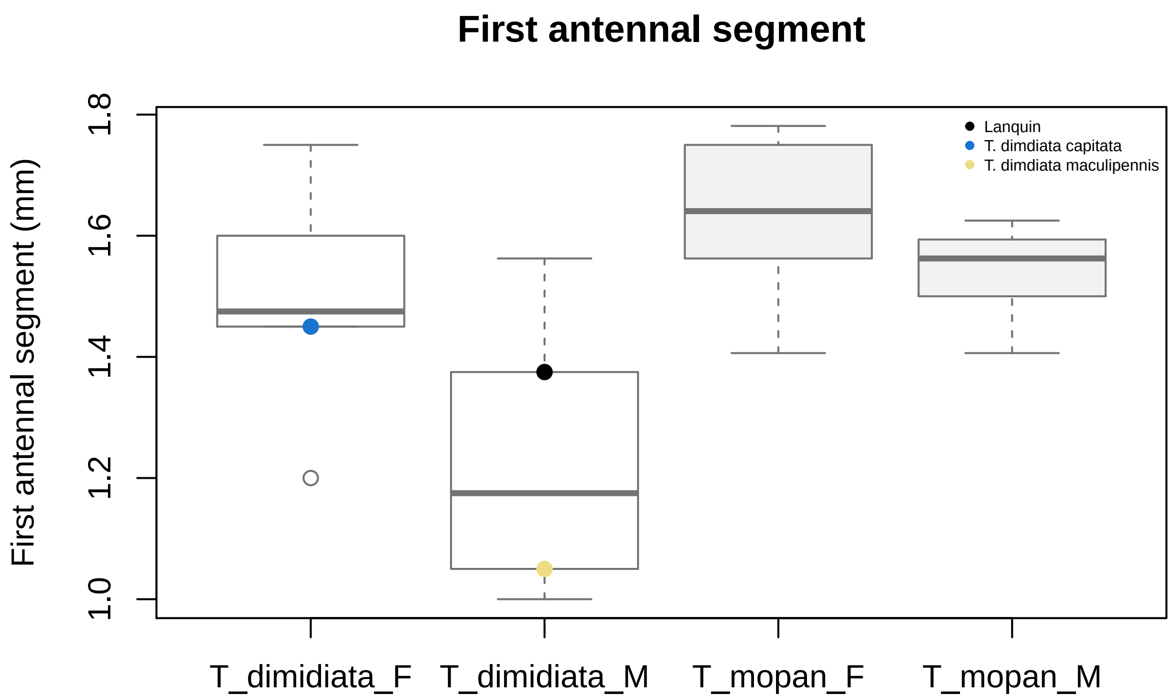


```
boxplot(data$A2 ~ data$Species, ylab ="Second antennal segment (mm)",
        border= c("gray45","gray45","gray45","gray45"),
        col=c("white", "white", "gray95", "gray95" ),
        main =  "Second antennal segment")
stripchart(Lanquin$A2, vertical=TRUE, add=TRUE,
           method="stack", col='black', pch=19, at = 2)
stripchart(Capitata$A2, vertical=TRUE, add=TRUE,
           method="stack", col='dodgerblue3', pch=19, at = 1)
stripchart(Macullipennis$A2, vertical=TRUE, add=TRUE,
           method="stack", col='lightgoldenrod', pch=19, at = 2)
legend("topright", legend = c("Lanquin", "T. dimdiata capitata",
                              "T. dimdiata maculipennis"),
       col = c('black','dodgerblue3','lightgoldenrod'),
       cex = 0.5, pch = 19, bty = "n")
```

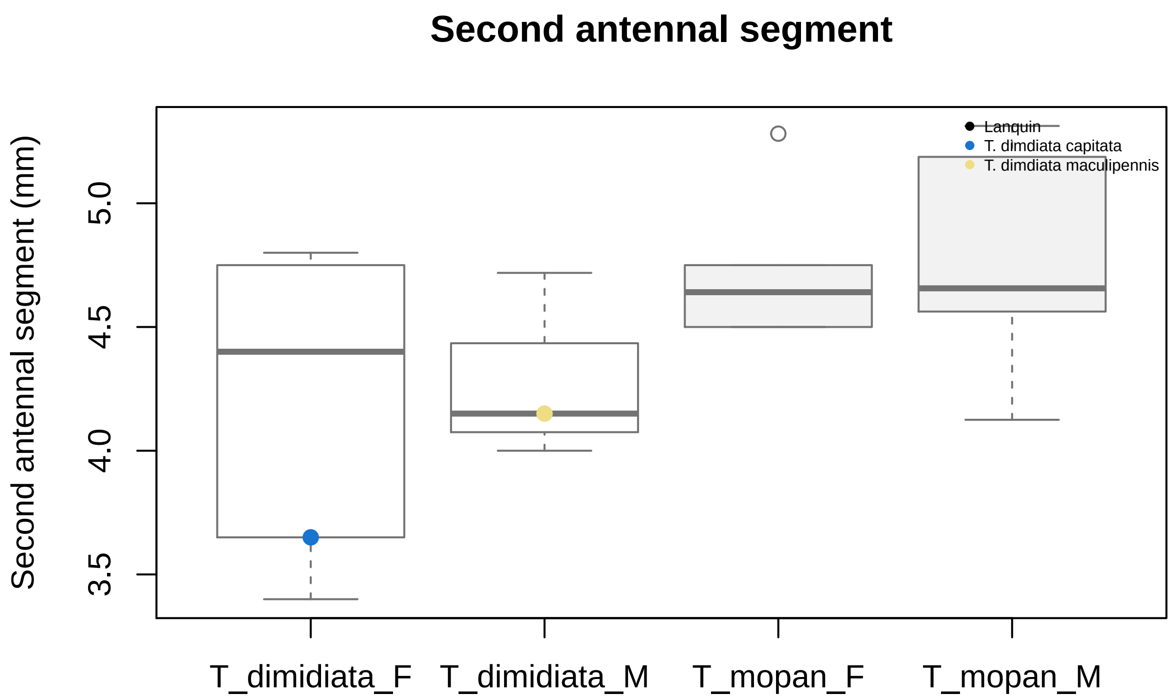


```r
boxplot(data$A3 ~ data$Species, ylab ="Third antennal segment (mm)",
        border= c("gray45","gray45","gray45","gray45"),
        col=c("white", "white", "gray95", "gray95" ),
        main =  "Third antennal segment")
stripchart(Lanquin$A3, vertical=TRUE, add=TRUE,
           method="stack", col='black', pch=19, at = 2)
stripchart(Capitata$A3, vertical=TRUE, add=TRUE,
           method="stack", col='dodgerblue3', pch=19, at = 1)
stripchart(Macullipennis$A3, vertical=TRUE, add=TRUE,
           method="stack", col='lightgoldenrod', pch=19, at = 2)
legend("topright", legend = c("Lanquin", "T. dimdiata capitata",
                              "T. dimdiata maculipennis"),
       col = c('black','dodgerblue3','lightgoldenrod'),
       cex = 0.5, pch = 19, bty = "n")
```

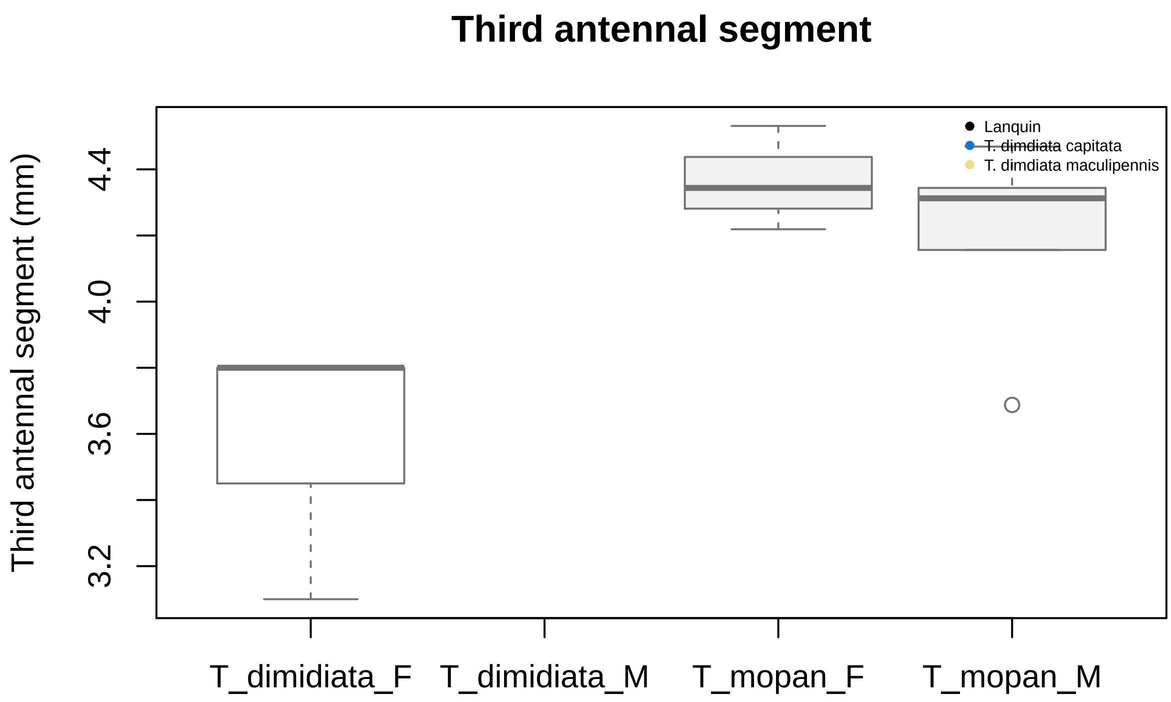


```
boxplot(data$BOS ~ data$Species, ylab ="Body of Scutellum (mm)",
        border= c("gray45","gray45","gray45","gray45"),
        col=c("white", "white", "gray95", "gray95" ),
        main =  "Body of Scutellum")
stripchart(Lanquin$BOS, vertical=TRUE, add=TRUE,
           method="stack", col='black', pch=19, at = 2)
stripchart(Capitata$BOS, vertical=TRUE, add=TRUE,
           method="stack", col='dodgerblue3', pch=19, at = 1)
stripchart(Macullipennis$BOS, vertical=TRUE, add=TRUE,
           method="stack", col='lightgoldenrod', pch=19, at = 2)
legend("topright", legend = c("Lanquin", "T. dimdiata capitata",
                              "T. dimdiata maculipennis"),
       col = c('black','dodgerblue3','lightgoldenrod'),
       cex = 0.5, pch = 19, bty = "n")
```

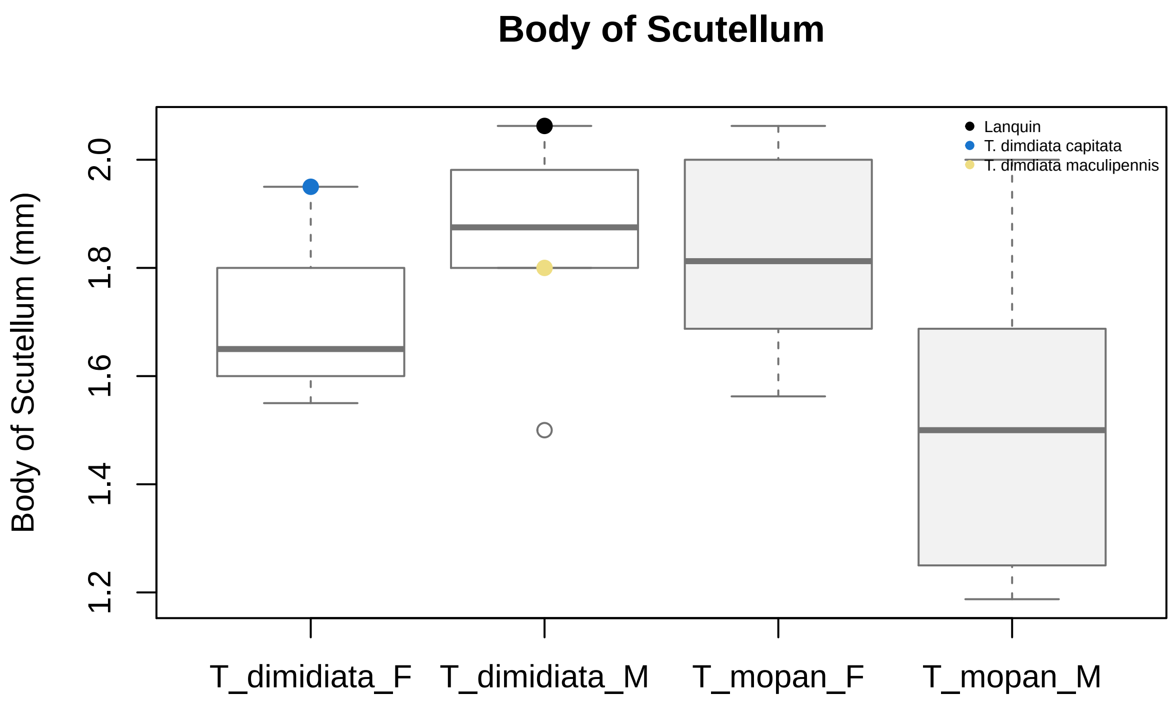


```r
boxplot(data$LA ~ data$Species, ylab ="Length of abdomen (mm)",
        border= c("gray45","gray45","gray45","gray45"),
        col=c("white", "white", "gray95", "gray95" ),
        main =  "Length of abdomen")
stripchart(Lanquin$LA, vertical=TRUE, add=TRUE,
           method="stack", col='black', pch=19, at = 2)
stripchart(Capitata$LA, vertical=TRUE, add=TRUE,
           method="stack", col='dodgerblue3', pch=19, at = 1)
stripchart(Macullipennis$LA, vertical=TRUE, add=TRUE,
           method="stack", col='lightgoldenrod', pch=19, at = 2)
legend("topright", legend = c("Lanquin", "T. dimdiata capitata",
                              "T. dimdiata maculipennis"),
       col = c('black','dodgerblue3','lightgoldenrod'),
       cex = 0.5, pch = 19, bty = "n")
```

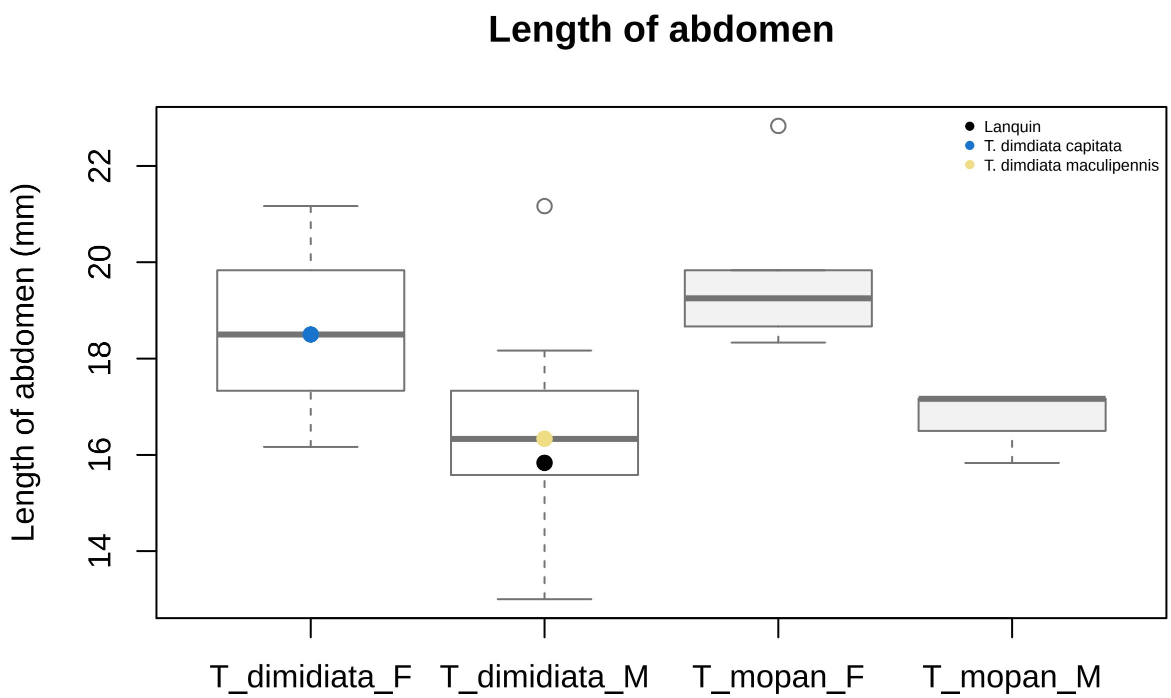


```
boxplot(data$R2 ~ data$Species, ylab ="Second visible segment of the labium (mm)",
        border= c("gray45","gray45","gray45","gray45"),
        col=c("white", "white", "gray95", "gray95" ),
        main =  "Second visible segment of the labium")
stripchart(Lanquin$R2, vertical=TRUE, add=TRUE,
           method="stack", col='black', pch=19, at = 2)
stripchart(Capitata$R2, vertical=TRUE, add=TRUE,
           method="stack", col='dodgerblue3', pch=19, at = 1)
stripchart(Macullipennis$R2, vertical=TRUE, add=TRUE,
           method="stack", col='lightgoldenrod', pch=19, at = 2)
legend("topright", legend = c("Lanquin", "T. dimdiata capitata",
                              "T. dimdiata maculipennis"),
       col = c('black','dodgerblue3','lightgoldenrod'),
       cex = 0.5, pch = 19, bty = "n")
```

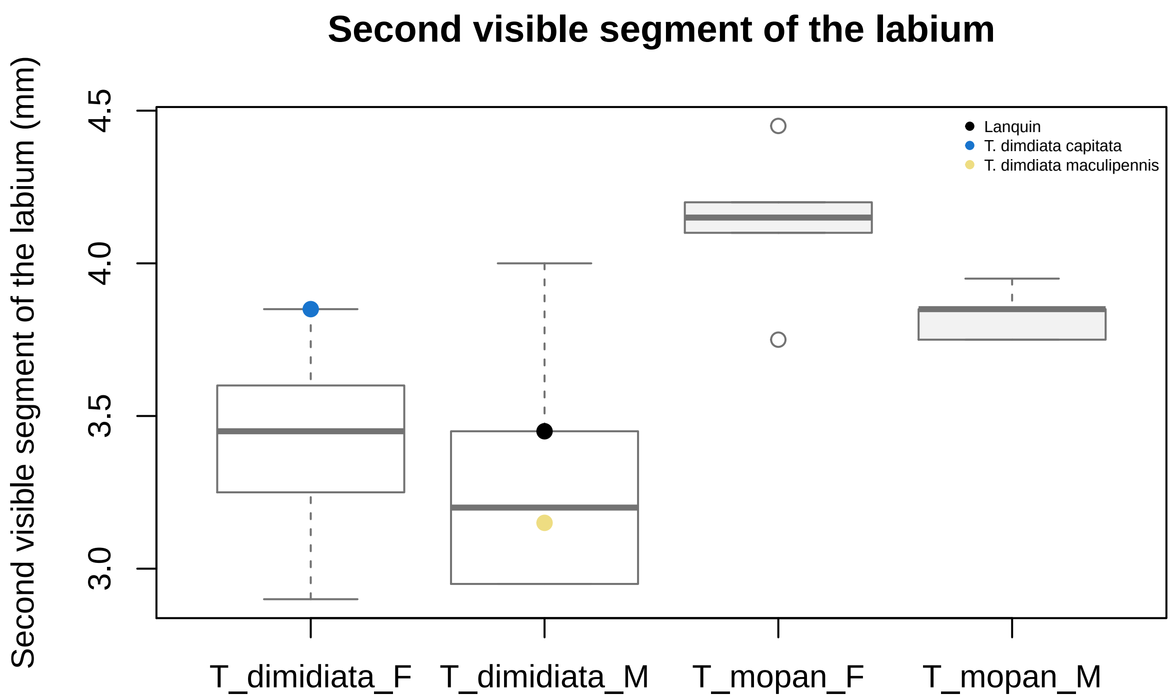


```r
boxplot(data$R1 ~ data$Species, ylab ="First visible segment of the labium (mm)",
        border= c("gray45","gray45","gray45","gray45"),
        col=c("white", "white", "gray95", "gray95" ),
        main =  "First visible segment of the labium")
stripchart(Lanquin$R1, vertical=TRUE, add=TRUE,
           method="stack", col='black', pch=19, at = 2)
stripchart(Capitata$R1, vertical=TRUE, add=TRUE,
           method="stack", col='dodgerblue3', pch=19, at = 1)
stripchart(Macullipennis$R1, vertical=TRUE, add=TRUE,
           method="stack", col='lightgoldenrod', pch=19, at = 2)
legend("topright", legend = c("Lanquin", "T. dimdiata capitata",
                              "T. dimdiata maculipennis"),
       col = c('black','dodgerblue3','lightgoldenrod'),
       cex = 0.5, pch = 19, bty = "n")
```

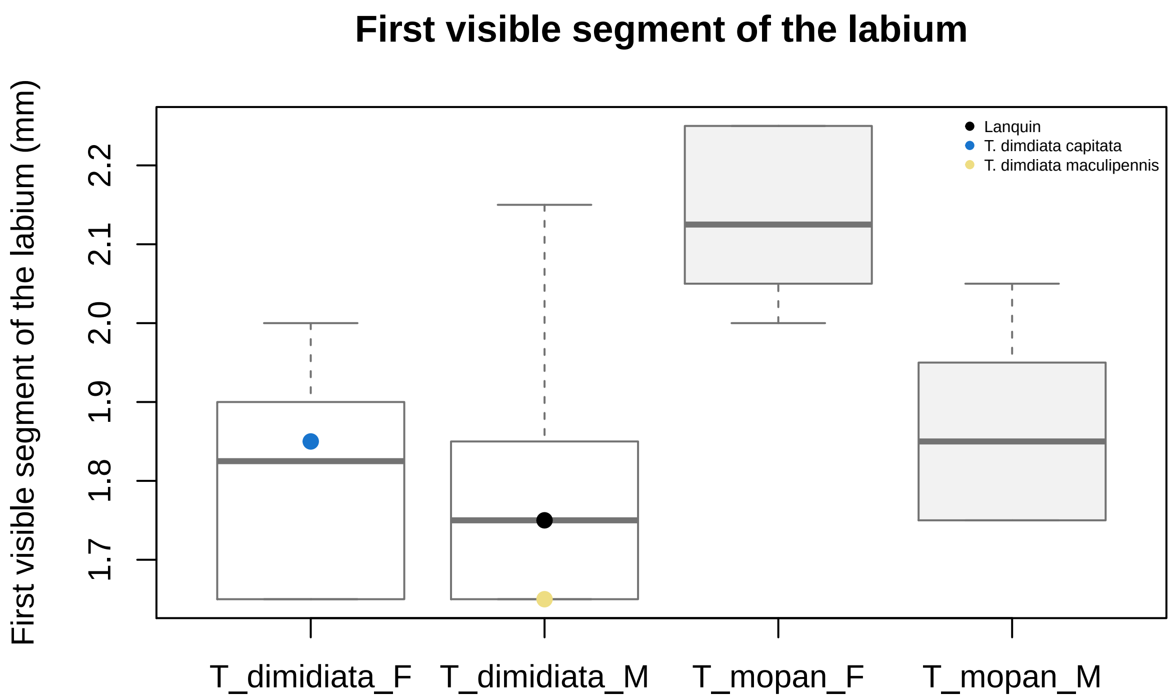


```
boxplot(data$R3 ~ data$Species, ylab ="Third visible segment of the labium (mm)",
        border= c("gray45","gray45","gray45","gray45"),
        col=c("white", "white", "gray95", "gray95" ),
        main =  "Third visible segment of the labium")
stripchart(Lanquin$R3, vertical=TRUE, add=TRUE,
           method="stack", col='black', pch=19, at = 2)
stripchart(Capitata$R3, vertical=TRUE, add=TRUE,
           method="stack", col='dodgerblue3', pch=19, at = 1)
stripchart(Macullipennis$R3, vertical=TRUE, add=TRUE,
           method="stack", col='lightgoldenrod', pch=19, at = 2)
legend("topright", legend = c("Lanquin", "T. dimdiata capitata",
                              "T. dimdiata maculipennis"),
       col = c('black','dodgerblue3','lightgoldenrod'),
       cex = 0.5, pch = 19, bty = "n")
```

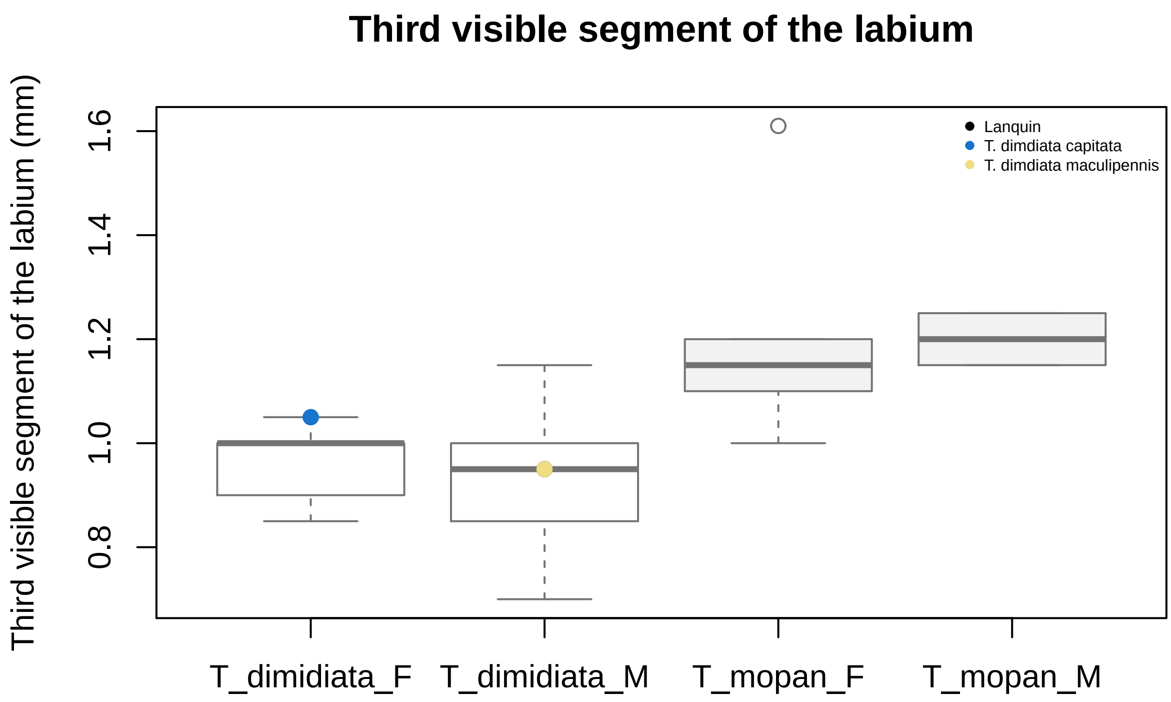


```
boxplot(data$S ~ data$Species, ylab ="Interocular region (mm)",
        border= c("gray45","gray45","gray45","gray45"),
        col=c("white", "white", "gray95", "gray95" ),
        main =  "Interocular region")
stripchart(Lanquin$S, vertical=TRUE, add=TRUE,
           method="stack", col='black', pch=19, at = 2)
stripchart(Capitata$S, vertical=TRUE, add=TRUE,
           method="stack", col='dodgerblue3', pch=19, at = 1)
stripchart(Macullipennis$S, vertical=TRUE, add=TRUE,
           method="stack", col='lightgoldenrod', pch=19, at = 2)
legend("topright", legend = c("Lanquin", "T. dimdiata capitata",
                              "T. dimdiata maculipennis"),
       col = c('black','dodgerblue3','lightgoldenrod'),
       cex = 0.5, pch = 19, bty = "n")
```

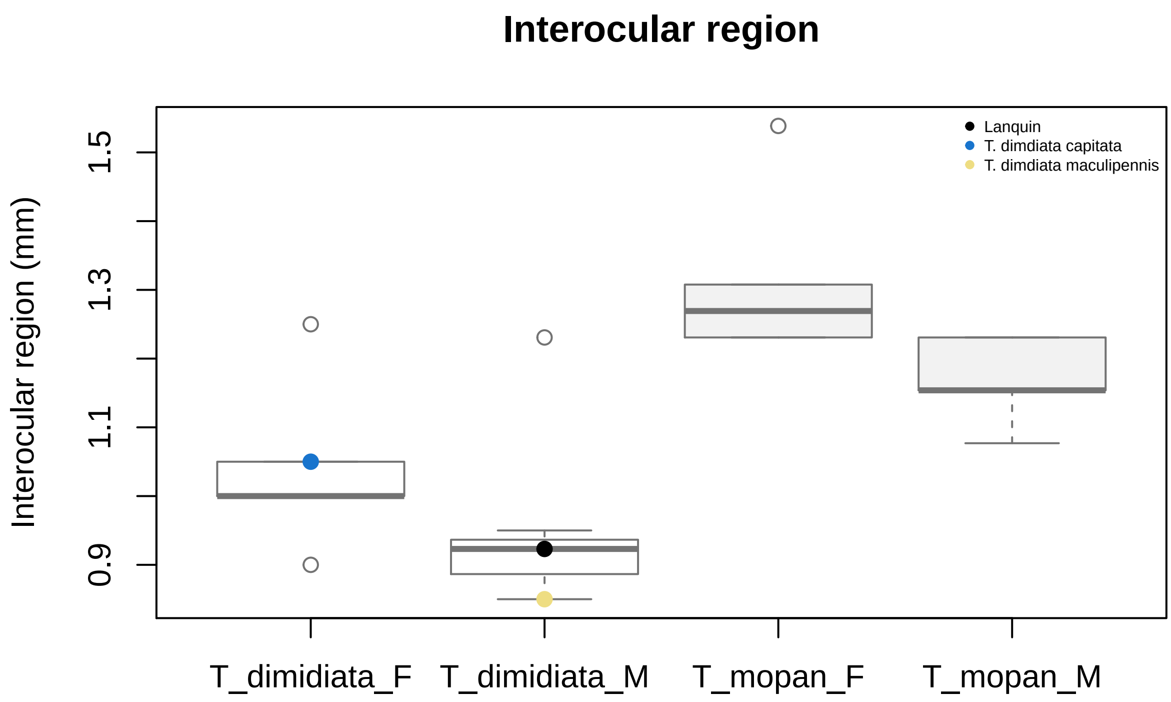


```
boxplot(data$Scut ~ data$Species, ylab ="Total legth of scutellum (mm)",
        border= c("gray45","gray45","gray45","gray45"),
        col=c("white", "white", "gray95", "gray95" ),
        main =  "Total legth of scutellum")
stripchart(Lanquin$Scut, vertical=TRUE, add=TRUE,
           method="stack", col='black', pch=19, at = 2)
stripchart(Capitata$Scut, vertical=TRUE, add=TRUE,
           method="stack", col='dodgerblue3', pch=19, at = 1)
stripchart(Macullipennis$Scut, vertical=TRUE, add=TRUE,
           method="stack", col='lightgoldenrod', pch=19, at = 2)
legend("topright", legend = c("Lanquin", "T. dimdiata capitata",
                              "T. dimdiata maculipennis"),
       col = c('black','dodgerblue3','lightgoldenrod'),
       cex = 0.5, pch = 19, bty = "n")
```

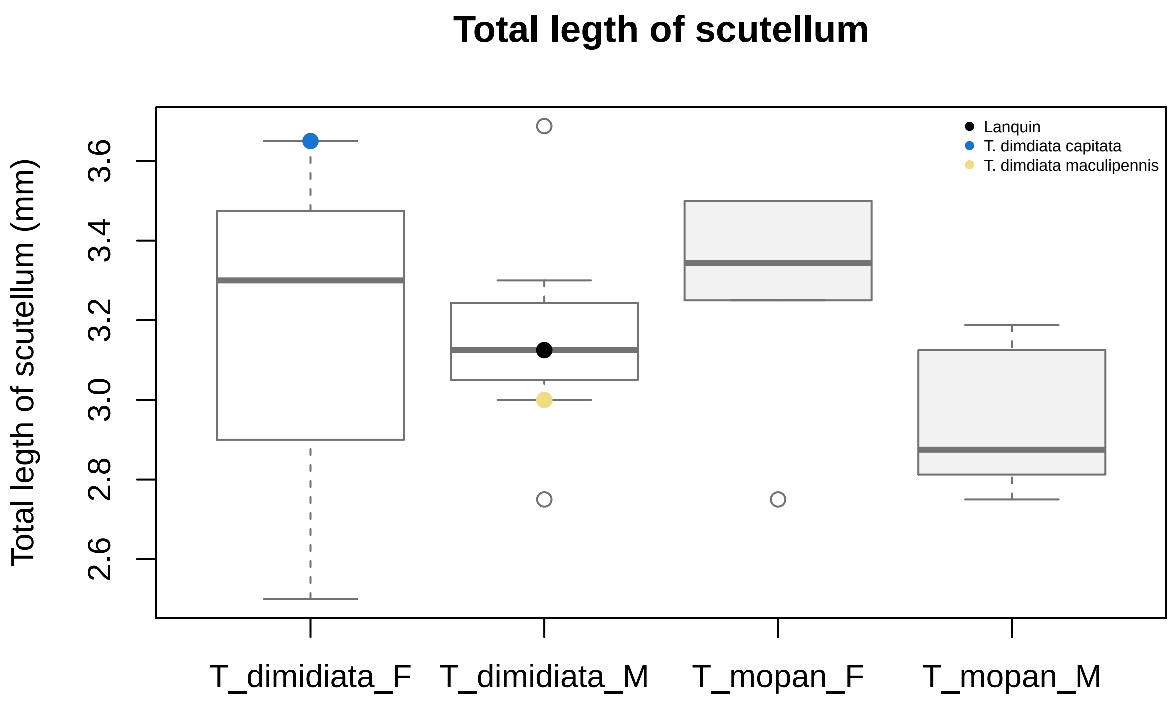
Total legth of scutellum
Total legth of scutellum (mm)
2.6
2.8
3.0
3.2
3.4
3.6
Lanquin
T. dimdiata capitata
T. dimdiata maculipennis
T_dimidiata_F
T_dimidiata_M
T_mopan_F
T_mopan_M
