# Supplementary material for: Description of Triatoma mopan sp. n. from a cave in Belize (Hemiptera, Reduviidae, Triatominae)
Source: Zookeys. 2018 Jul 17;(775):69–95. doi: 10.3897/zookeys.775.22553 (PMC6058004; doi:10.3897/zookeys.775.22553)
Supplement: Supplementary material 2 — T. mopan description - R codes. [file zookeys-775-069-s002.pdf]

T. mopan description - R codes

Silvia Justi

February 7, 2018

Libraries used

```
library(ape)
```

CytB and ITS-2 alignments

```
cytb<-read.FASTA("Cyt_b__mopan.fst")
```

```
its2<-read.FASTA("its2_mopan_align.fst")
```

Calculating pairwise k2p distances. This model was used to make the distances comparable to previously published data.

```
dist_cytb <- dist.dna(cytb, model = "k80", as.matrix = TRUE, pairwise.deletion = T)
```

Ordering the distances from smallest to largest to plot and visualize it. Red dotted line corresponds to a distance of 2%.

```
T_mopan <- subset(dist_cytb, select = "T_mopan_1")
T_mopan_sorted <- as.matrix(T_mopan[order(T_mopan[, 1]), ])
plot(T_mopan_sorted, pch = 1, main = "K2p pairwise distances", xaxt = "n", xlab = "",
    ylab = "k2p distance")
axis(1, at = 1:nrow(T_mopan_sorted), labels = row.names(T_mopan_sorted), las = 2,
    cex.axis = 0.5)
abline(h = 0.02, col = "red", lty = 3)
```

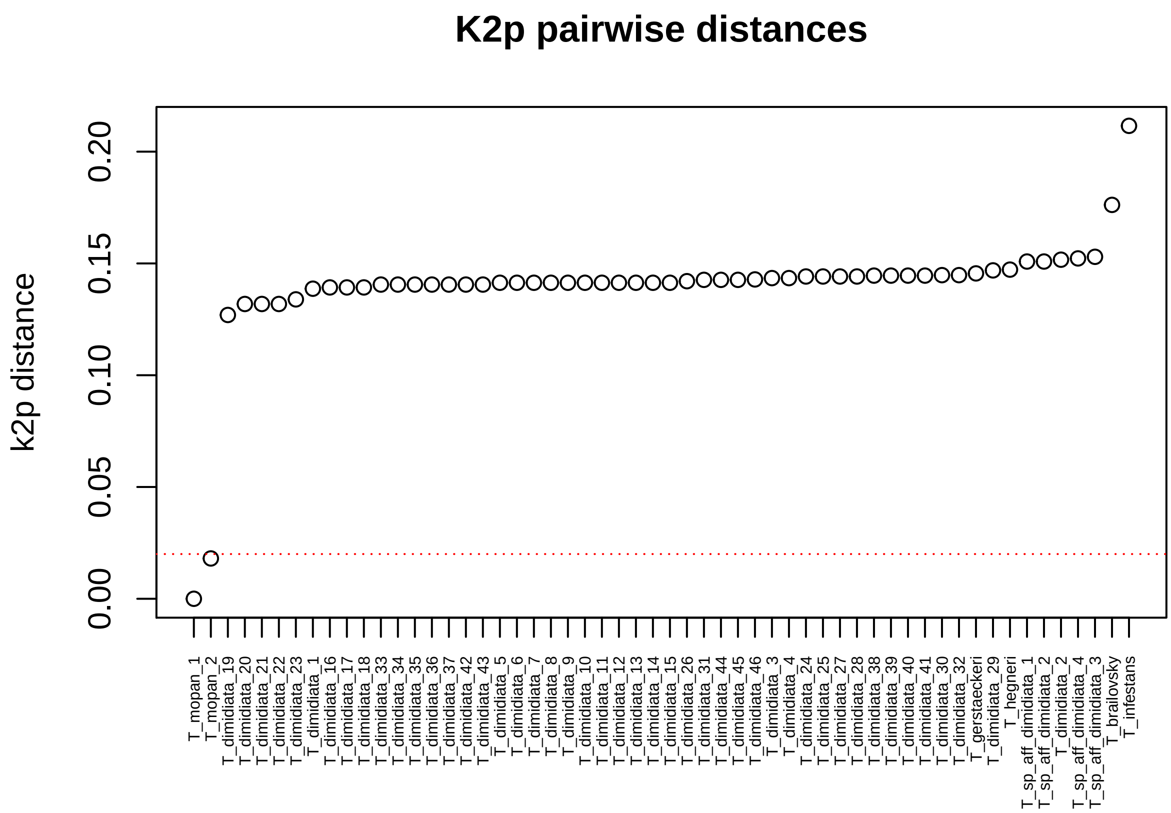


```
dist_its2 <- dist.dna(its2, model = "k80", as.matrix = TRUE, pairwise.deletion = T)
```

Ordering the distances from smallest to largest to plot and visualize it. Red dotted line corresponds to a distance of 2%.

```
T_mopan_its2 <- subset(dist_its2, select = "T_mopan_1")
T_mopan_its2sorted <- as.matrix(T_mopan_its2[order(T_mopan_its2[, 1]), ])
plot(T_mopan_its2sorted, pch = 1, main = "K2p pairwise distances", xaxt = "n",
    xlab = "", ylab = "k2p distance")
axis(1, at = 1:nrow(T_mopan_its2sorted), labels = row.names(T_mopan_its2sorted),
    las = 2, cex.axis = 0.5)
abline(h = 0.02, col = "red", lty = 3)
```

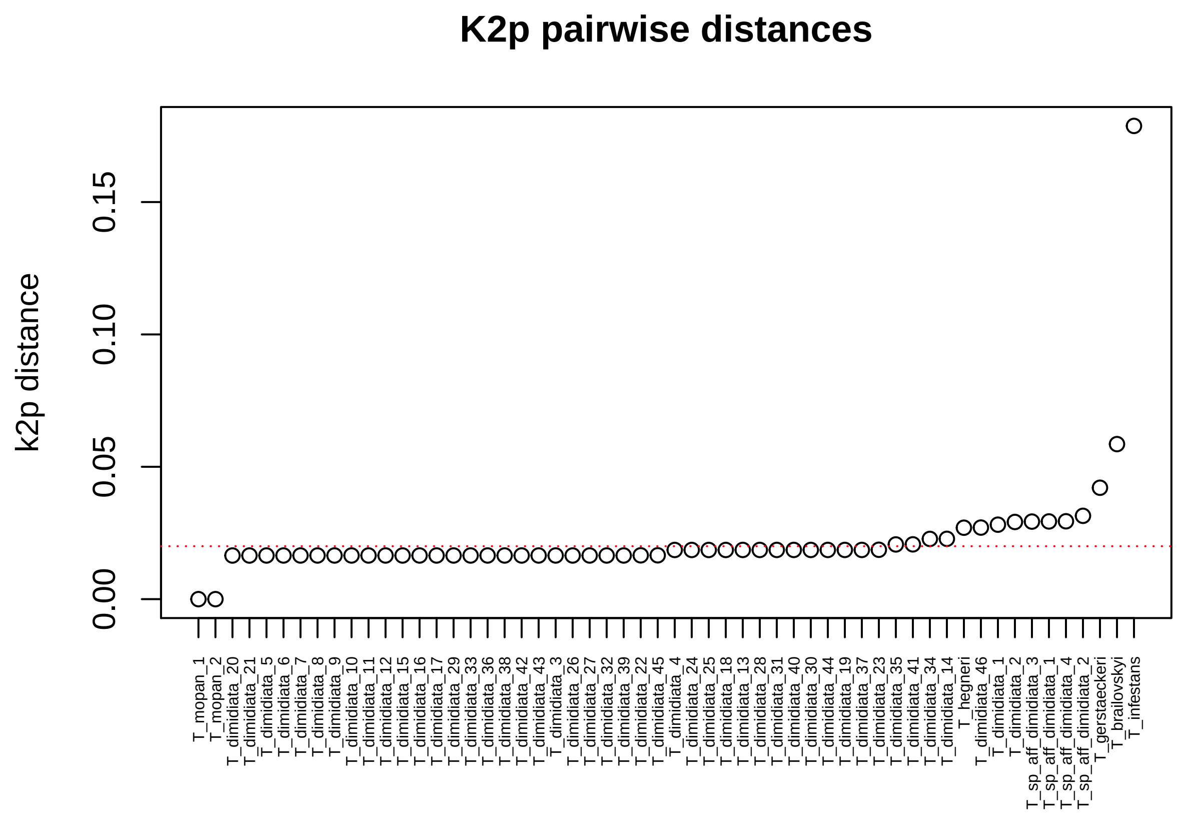

Reading as displaying the phylogenies reconstructed using PhyML

```
cytb_tree<-read.tree("cytb_mopan.tre")
plot(cytb_tree, type = "phylogram", use.edge.length = TRUE,
     node.pos = 1, show.tip.label = TRUE,
     show.node.label = TRUE,
     edge.color = "black",
     edge.width = 1,
     edge.lty = 1,
     font = 1,
     cex = 0.5,
     no.margin = TRUE,
     root.edge = TRUE,
     label.offset = 0,
     underscore = FALSE,
     x.lim = NULL, y.lim = NULL,
     direction = "rightwards",
     lab4ut = NULL, tip.color = "black", plot = TRUE,
     rotate.tree = 0, open.angle = 0, node.depth = 1,
     align.tip.label = FALSE)
```

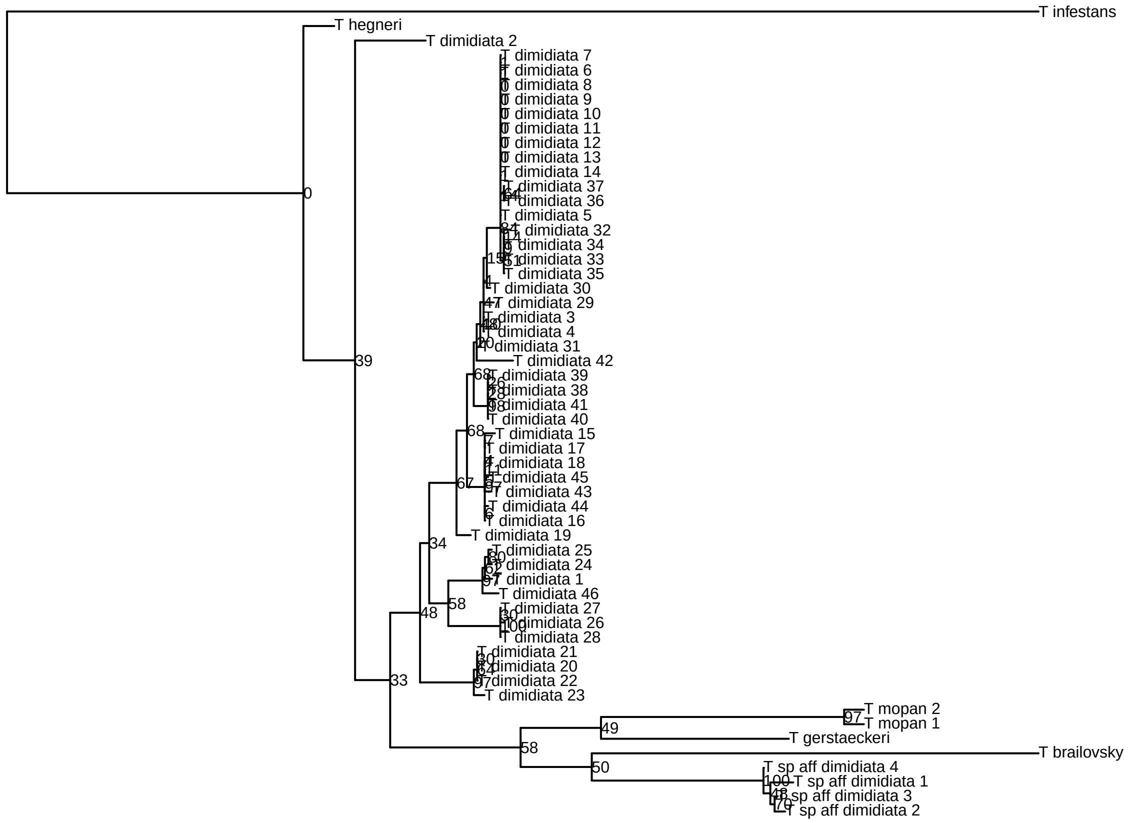


```r
its2_tree<-read.tree("its2_mopan.tre")
plot(its2_tree, type = "phylogram", use.edge.length = TRUE,
     node.pos = 1, show.tip.label = TRUE,
     show.node.label = TRUE,
     edge.color = "black",
     edge.width = 1,
     edge.lty = 1,
     font = 1,
     cex = 0.5,
     no.margin = TRUE,
     root.edge = TRUE,
     label.offset = 0,
     underscore = FALSE,
     x.lim = NULL, y.lim = NULL,
     direction = "rightwards",
     lab4ut = NULL, tip.color = "black", plot = TRUE,
     rotate.tree = 0, open.angle = 0, node.depth = 1,
     align.tip.label = FALSE)
```

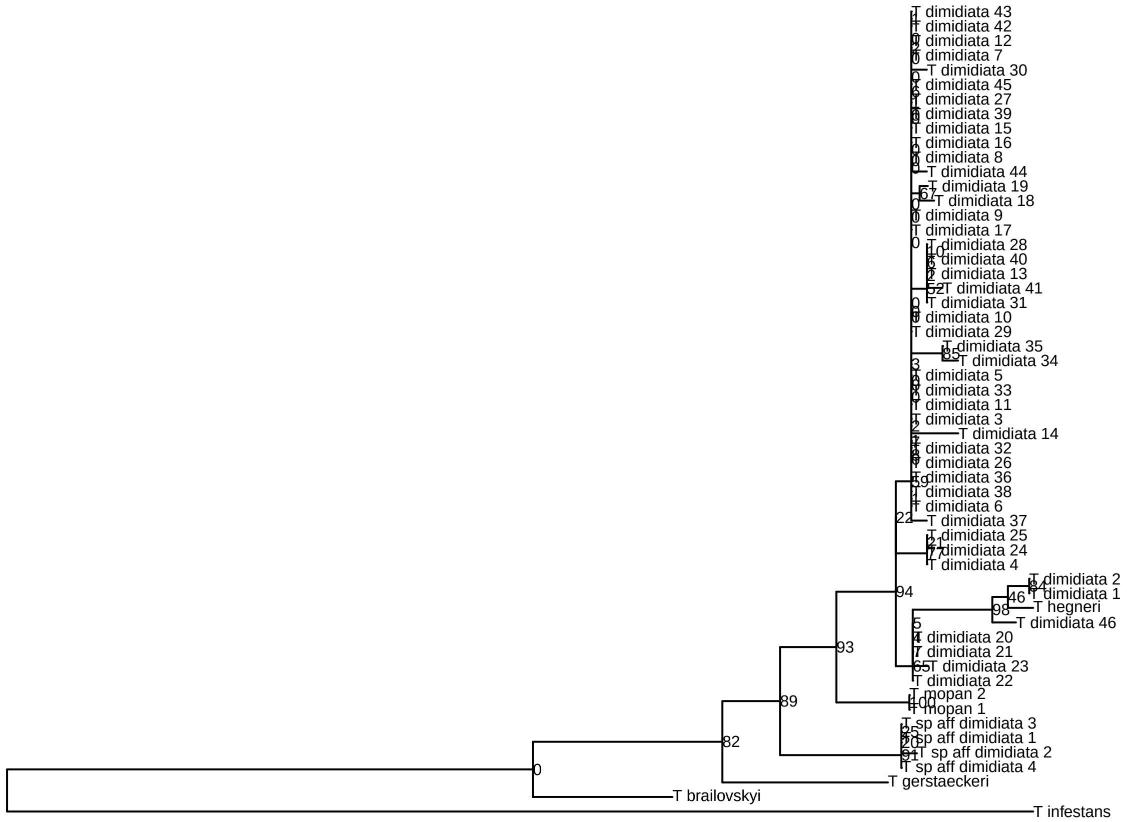
T dimidiata 43
T dimidiata 42
T dimidiata 12
T dimidiata 7
T dimidiata 30
T dimidiata 45
T dimidiata 27
T dimidiata 39
T dimidiata 15
T dimidiata 16
T dimidiata 8
T dimidiata 44
T dimidiata 19
T dimidiata 18
T dimidiata 9
T dimidiata 17
T dimidiata 28
T dimidiata 40
T dimidiata 13
T dimidiata 41
T dimidiata 31
T dimidiata 10
T dimidiata 29
T dimidiata 35
T dimidiata 34
T dimidiata 5
T dimidiata 33
T dimidiata 11
T dimidiata 3
T dimidiata 14
T dimidiata 32
T dimidiata 26
T dimidiata 36
T dimidiata 38
T dimidiata 6
T dimidiata 37
T dimidiata 25
T dimidiata 24
T dimidiata 4
T dimidiata 2
T dimidiata 1
T hegneri
T dimidiata 46
T dimidiata 20
T dimidiata 21
T dimidiata 23
T dimidiata 22
T mopan 2
T mopan 1
T sp aff dimidiata 3
T sp aff dimidiata 1
T sp aff dimidiata 2
T sp aff dimidiata 4
T gerstaeckeri
T brailovskyi
T infestans
22
94
98
46
93
89
82
0
100
